# Lateral Transfer of a Lectin-Like Antifreeze Protein Gene in Fishes

**DOI:** 10.1371/journal.pone.0002616

**Published:** 2008-07-09

**Authors:** Laurie A. Graham, Stephen C. Lougheed, K. Vanya Ewart, Peter L. Davies

**Affiliations:** 1 Department of Biochemistry, Queen's University, Kingston, Ontario, Canada; 2 Department of Biology, Queen's University, Kingston, Ontario, Canada; 3 NRC Institute for Marine Biosciences, Halifax, Nova Scotia, Canada; Center for Genomic Regulation, Spain

## Abstract

Fishes living in icy seawater are usually protected from freezing by endogenous antifreeze proteins (AFPs) that bind to ice crystals and stop them from growing. The scattered distribution of five highly diverse AFP types across phylogenetically disparate fish species is puzzling. The appearance of radically different AFPs in closely related species has been attributed to the rapid, independent evolution of these proteins in response to natural selection caused by sea level glaciations within the last 20 million years. In at least one instance the same type of simple repetitive AFP has independently originated in two distant species by convergent evolution. But, the isolated occurrence of three very similar type II AFPs in three distantly related species (herring, smelt and sea raven) cannot be explained by this mechanism. These globular, lectin-like AFPs have a unique disulfide-bonding pattern, and share up to 85% identity in their amino acid sequences, with regions of even higher identity in their genes. A thorough search of current databases failed to find a homolog in any other species with greater than 40% amino acid sequence identity. Consistent with this result, genomic Southern blots showed the lectin-like AFP gene was absent from all other fish species tested. The remarkable conservation of both intron and exon sequences, the lack of correlation between evolutionary distance and mutation rate, and the pattern of silent vs non-silent codon changes make it unlikely that the gene for this AFP pre-existed but was lost from most branches of the teleost radiation. We propose instead that lateral gene transfer has resulted in the occurrence of the type II AFPs in herring, smelt and sea raven and allowed these species to survive in an otherwise lethal niche.

## Introduction

Acquisition of a new gene/trait typically arises from gene duplication and divergence [1]. A classic example of this gradual process is the evolution of a set of pancreatic serine proteases, specifically trypsin, chymotrypsin and elastase, from a common precursor [2], [3]. These paralogs have the same three-dimensional fold and operate by the same enzymatic mechanism, but cleave proteins after different amino acids. The opportunities to short-circuit this process and pass a gene between species by horizontal or lateral gene transfer (LGT) would seem extremely limited, and are largely restricted to prokaryotes. In some bacteria there are established routes (conjugation, transduction and transformation) for the exchange of DNA between strains/species, subject to the strictures of the restriction/modification system in the recipient host. LGT becomes particularly evident where the acquisition of the transferred gene confers a selective advantage on the host, as for example in antibiotic resistance [4], [5]. Here, there is the opportunity to acquire a new gene type within one generation rather than by gradual evolution.

In eukaryotes there is no established mechanism for transferring intact genes between species, although retrovirally processed sequences have been transferred [6]. There is also the added difficulty that genes are packaged within organelles (principally the nucleus) and are therefore less accessible than genes in bacteria. Moreover, in higher eukaryotes there is an additional barrier to transmission in that only LGT to germ-line cells would be passed on. Thus, prior to this report there was no well documented report of a standard eukaryotic gene being passed into or between vertebrate species. Here we provide the first such evidence for LGT in vertebrates. As with antibiotic resistance in bacteria, it has come to light because of the selective advantage the gene confers to a host under intense selective pressure.

**Figure 1:**
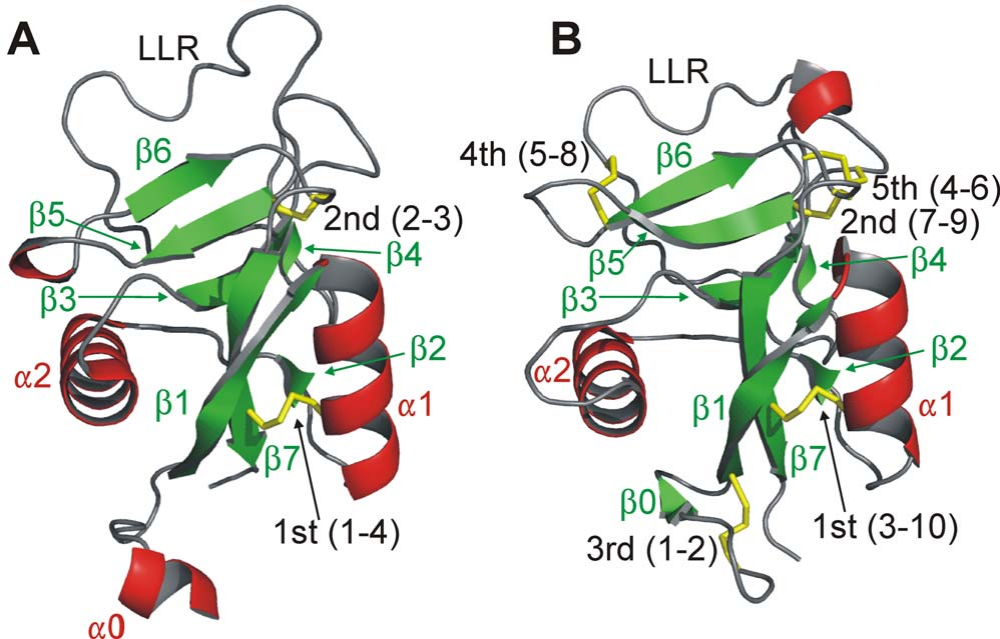
Antifreeze protein - lectin structural comparisons. (A) Rat mannose-binding protein structure (PDB code: 1KWT) and (B) Herring AFP structure (PDB code: 2PY2) showing the location of the 5^th^ disulfide bridge that is peculiar to the type II AFPs. Secondary structure elements (red – helix; green – beta-strand; grey – loop) are numbered from the N terminus. Disulfide bonds are shown in yellow with the linkages of numbered cysteines indicated in brackets. LLR is the long loop region.

The gene in question codes for a type II antifreeze protein (AFP), one of five distinct types that have appeared in fishes. These AFPs stop the growth of seed ice crystals by a surface adsorption-inhibition mechanism and thereby help fish resist freezing in icy seawater [7]. Type II AFPs are homologs (paralogs) of the sugar-binding domain of Ca^2+^-dependent (C-type) lectins [8]. In C-type lectins [9], [10] such as the rat mannose-binding protein (now named mannan-binding lectin) (Figure 1A), one of the calcium ions is an integral part of the sugar-binding site and makes direct contact with the ligand [11]. Herring and rainbow smelt AFPs require Ca^2+^ for binding to ice [8], [12]. Again, this metal ion is thought to play a central role in ligand binding because substitution of Ca^2+^ with other divalent metal ions alters both the antifreeze activity and ice crystal morphology [13]. X-ray crystallography has shown that herring AFP [14] has same fold as rat mannose-binding protein (Figure 1A,B). The more divergent sea raven AFP [15], which is 40% identical to herring and smelt AFPs, is not Ca^2+^-dependent. Nonetheless, solution structure determination has shown that it too has the same fold as rat mannose-binding protein [16].

**Figure 2:**
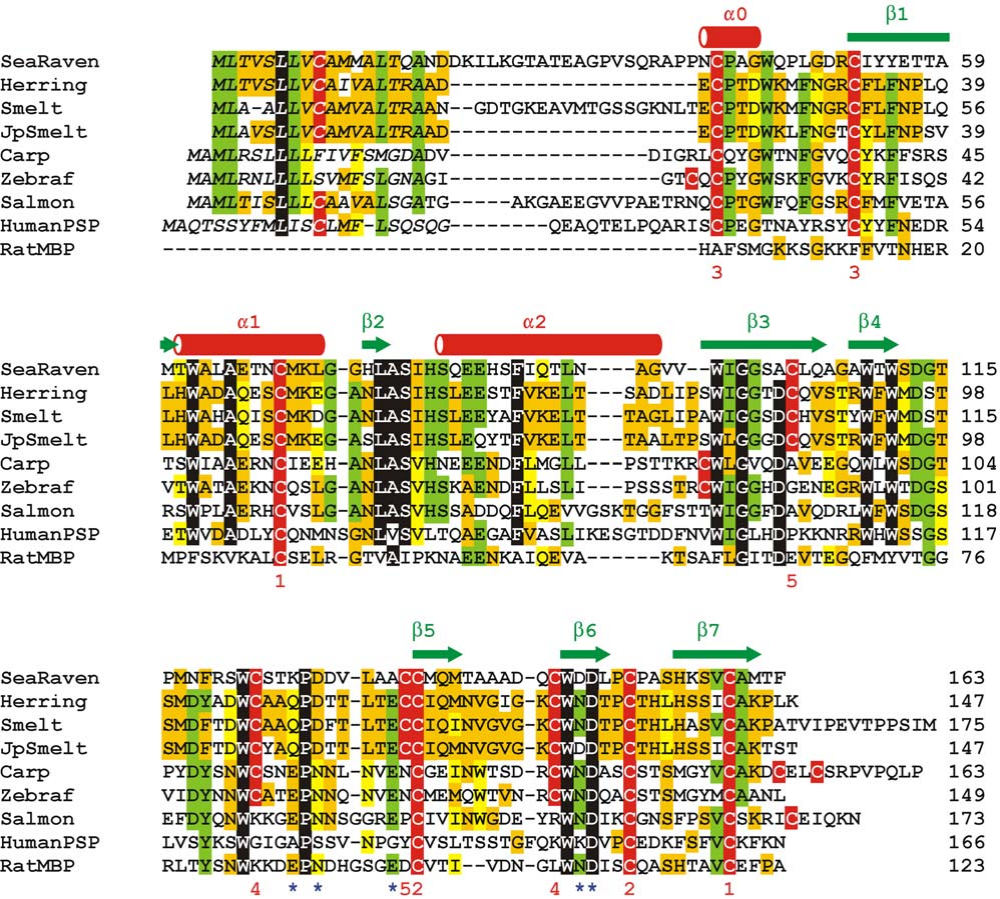
Antifreeze protein - lectin alignments. C-type lectin homolog alignment showing the cysteine pair that makes up the 5^th^ disulfide bond is common to the type II AFPs but is missing in other lectins from the database. Sequences aligned are type II AFPs from the sea raven, *Hemitripterus americanus*, (SeaRaven, GenBank #AAA49617); the Atlantic herring, *Clupea harengus*, (Herring, GenBank # AAY60837); the rainbow smelt, *Osmerus mordax*, (Smelt, GenBank #AAA49442); the Japanese smelt, *Hypomesus nipponensis*, (JpSmelt (Yamashita et al. 2003)); single lectin domain proteins from the common carp, *Cyprinus carpio*, (Carp, GenBank #BAA95671); the zebrafish, *Danio rerio*, (Zebraf, GenBank # XM_001337634); and the Atlantic salmon, *Salmo salar*, (Salmon, GenBank #AAO43604); as well as the human pancreatic stone protein (PSP, GenBank #NP_002900) and the C-type lectin domain from the rat mannose-binding protein (RatMBP, GenBank #AAA98781). Secondary structure elements from the rat mannose-binding protein (see Figure 1) are displayed above the alignment. Colour is used to indicate conservation of the residues between the 9 sequences with matches of 8–9 in black, 6–7 in green, and 3–5 in ochre or yellow. Cysteines are coloured red and their pairings are indicated by numbers under the alignment. Sequences are numbered from the start of the signal polypeptide (in italics) except for the rat mannose-binding protein where numbering starts at the beginning of the domain. Residues in the herring and rat mannose-binding protein that are involved in Ca^2+^ coordination are indicated by blue stars below the sequence alignment.

The structural feature that the three type II AFPs share, and which distinguishes them from all other C-type lectin domains, is that they have ten cysteines forming five disulfide bridges in identical positions (Figure 2). Most C-type lectins have two or three of the disulfide bridges [9], [10]. One of the two invariant bridges found in all C-type lectins with a long loop region links the first helix (α1) to the last β-strand (β7) (Figure 1A,B). The other links the start of β5 to the loop between β6 and β7. The 3^rd^ disulfide bridge occurs within the N-terminal extension that is missing from some lectins. A 4^th^ bridge linking the long loop region between β4 and β5 to the start of β6 is comparatively rare but is seen, for example, in some lectins from carp and zebrafish (Figure 2). However, the 5^th^ bridge, linking the loop after β3 to the first Cys of the pair found at the beginning of β3, is peculiar to the type II AFPs.

The independent gain of a disulfide bridge in exactly the same place on three separate occasions seems unlikely. In light of this, Liu et al. [14] have proposed that the type II AFPs are derived from a ten-Cys lectin isoform that preexisted in the ancestor to most fishes, but has subsequently been lost from all other branches. We have researched this possibility and find that the evidence, particularly for the herring and smelt AFPs, is overwhelmingly in favour of a different mechanism: LGT. The remarkable conservation of the protein sequences, the unexplained conservation of the intron sequences, the lack of correlation between evolutionary distance and mutation rate, and the pattern of silent vs non-silent codon changes all point to lateral transfer of the gene.

## Methods

Unpublished sequences have been deposited in GenBank with the following accession numbers: DQ008165 (Prp8 genomic sequence from rainbow smelt), DQ008166 (Prp8 genomic sequence from Atlantic herring), DQ004949 (AFP genomic sequence from rainbow smelt), DQ003023 (AFP genomic sequence from Atlantic herring).

### Isolation of genomic and gene-specific DNAs

Genomic DNAs were isolated from either testes (bowfin *Amia calva*, rainbow trout *Oncorhynchus mykiss*, Atlantic cod *Gadus morhua*, sea raven *Hemitripterus americanus*, yellow perch *Perca flavescens*, winter flounder *Pseudopleuronectes americanus*), liver (Atlantic herring *Clupea harengus*, rainbow smelt *Osmerus mordax*), muscle (Pacific herring *Clupea pallasi*, cisco *Coregonus artedi*) or whole fish (zebrafish *Danio rerio*) [17]. All fishes were caught off the Atlantic coast of Canada except the following: rainbow trout (Denmark), zebrafish (local pet store), bowfin, yellow perch and cisco (Lake Ontario), Pacific herring (Pacific coast of Canada).

The primers used to amplify the AFP gene sequences are as follows; rainbow smelt, upstream of the start codon 5′-CAACAGGCTGAAATTGTGCAGACA-3′, ending on the stop codon 5′-TCACATGATTGATGGTGGTGTCAC-3′ and Atlantic herring, upstream of the start codon 5′-CTAAAGGGAAGACAGAGGCAACAG-3′, downstream of the stop codon 5′-TGATTGATGGTGGTGGATGCCTCT-3′. Approximately 200 ng of genomic DNA was amplified using the Expand High-Fidelity PCR system (Roche, Penzberg, Germany). The DNA was denatured for 10 min at 95°C, reagents were added at 80°C, and 30 cycles of PCR were done as follows; 95°C for 1 min, 60°C for 1 min and 72°C for 3 min with 5 sec/cycle added starting at cycle 11, with a final extension of 7 min at 72°C. Products were subcloned into the pCR2.1-TOPO vector (Invitrogen, Carlsbad, CA, U.S.A.) and both strands were sequenced using vector and internal primers.

The primers used to amplify a portion of 16S rDNA from Atlantic herring and rainbow smelt are as follows; 5′-TGAAGACCTGTATGAATGG-3′ and 5′-TTGAACAAACGAACCCTTA-3′. The amplification and subcloning were done as described above but using Taq (Fermentas International, Burlington, ON, Canada) and an annealing temperature of only 50°C.

The primers used to amplify a portion of the Prp8p gene correspond to exonic sequences conserved between zebrafish and pufferfish. They are numbered sequentially from outermost to innermost; #1 sense 5′-CAGCCTGTGAAGGTGCGTGTGTC-3′, #2 sense 5′-TTCCGCTCTTTCAAGGCCACCAA-3′, #3 sense 5′-GGCATGTACCGCTACAAGTACAA-3′, #1 antisense 5′-CTTCCAAGGAATGTTGGCCTTCCA-3′, #2 antisense 5′-CCGTGTTGGTCCACCAGTCAGCTTT-3′, #3 antisense 5′-GAGATGCTTCAGGTCTTTGCACAT-3′. The rainbow smelt sequence was obtained using primers #1 sense and antisense. The Atlantic herring sequence was obtained in two overlapping segments using #2 sense with #3 antisense and #3 sense with #2 antisense. The amplification, subcloning and sequencing were done as for the AFP genes except that 1 µL of the first PCR reaction was reamplified for 25 cycles and an annealing temperature of 56°C was used.

### Phylogenetic analyses

The evolutionary affinities of the lectin-like AFPs were inferred using both Bayesian and parsimony approaches on the amino acid alignment. Human pancreatic stone protein (PSP) was used as the out-group for both. For Bayesian analysis, the aamodelpr = mixed option was used, where the Markov chain samples each of nine models of evolution according to its probability [18]. Two simultaneous analyses of 1,000,000 generations were run and sampled every 100 generations, until the Potential Scale Reduction Factors [19] for all parameters were very close to one (to the second decimal). Effective sample sizes for all parameters were estimated using TRACER [20] and were all substantially greater than 100, implying effective sampling of the posterior distribution of all parameters. For parsimony analysis, we first performed an exhaustive search in PAUP* [21] with gaps treated as a 21st amino acid, and then evaluated support for the resulting topology using a bootstrap analysis with 1000 pseudoreplicates and ten random additions per replicate.

The phylogenetic relationships between teleosts accepted here are those established by Miya et al. [22] based on complete mitochondrial genome sequences. However, to orient other intermediate species, particularly AFP-producing ones, to the phylogeny, Bayesian analysis was performed on an alignment of a portion of the 16S rDNA region, corresponding to bases 3081 to 3532 of zebrafish 16S rDNA (AC024175). All sequences were obtained from GenBank, except those obtained above for Atlantic herring and rainbow smelt, and the species not noted elsewhere which include the Japanese pilchard *Sardinops melanostictus*, common carp *Cyprinus carpio*, smooth lumpsucker *Aptocyclus ventricosus*, longhorn sculpin *Myoxocephalus octodecemspinosus*, Antarctic eelpout *Lycodichthys dearborni*, Antarctic toothfish *Dissostichus mawsoni*, dark-banded fusilier *Pterocaesio tile*, bastard halibut *Paralichthys olivaceus* and masked triggerfish *Sufflamen fraenatus*. The.time reversible+I+G model of evolution was selected from among 24 possible models using the Akaike Information Criterion (MrModelTest,) [23]. A Bayesian tree was generated using the program MrBayes [24], with two independent runs each with 1,000,000 generations of MCMC simulations (until the standard deviation of the split frequencies of the two runs was less than 0.01). Trees were sampled every 100 generations beginning at 250,000 generations.

### Bioinformatic analyses

Database searches were done with the complete sequences of rainbow smelt, herring and sea raven AFPs. The protein sequences were used in both protein-protein, position-specific iterated and translated BLAST searches (using default parameters) depending on the database searched (http://www.ncbi.nlm.nih.gov/). The cDNA and genomic sequences were used in BLASTn searches using both default parameters (word size = 11, expect threshold = 10, match = 2, mismatch = −3, gap existence = −5, gap extension = −2, filter and mask on) and with altered parameters (word size = 7, filter and mask off). The following GenBank databases were searched: non redundant, EST, genome survey sequence, high-throughput genomic sequence, patent, whole genome shotgun, sequence tagged site and environmental sequences. Searches of the nr and EST databases included 1) all organisms; 2) just bony fishes (taxid∶32443); 3) everything but bony fishes; Species-specific search were performed, as above, on the nr, EST, high-throughput genomic sequence, whole genome shotgun and trace archives for zebrafish (taxid∶7955), *Takifugu rubripes* (taxid∶31033) and *Tetraodon nigroviridis* (taxid∶99883). The medaka (*Oryzias latipes*) BLAST was done at http://dolphin.lab.nig.ac.jp/medaka/ using BLASTn (word size = 7) and tBLASTn (word size = 3, BLOSUM62 scoring matrix) with the filter off and gaps allowed.

The individual intron and exon sequences of herring AFP, as well as intron 2 of smelt AFP, were used for nucleotide-nucleotide searches (BLASTn) against teleost fish sequences (taxid∶32443) in the non-redundant database as above.

### Analysis of synonymous and non-synonymous substitutions and codon bias

The ratio of non-synonymous substitutions per non-synonymous site to synonymous substitutions per synonymous site (*d_N_*/*d_S_*) was calculated using the SNAP tool [25]. The portion of AFP sequence compared extends from the first residue of the mature herring AFP (ECP…) to the last residue of the seventh beta strand (…CAK, Figure 2). A section of the sea raven sequence that could not be unambiguously aligned (AGVV in second helix, Figure 2) was excluded in comparisons with this sequence. The portion of Prp8p coding sequence compared corresponds to the overlapping region between the sections of the rainbow smelt and herring sequences cloned in this study.

The codon usage, effective number of codons (EN_c_), and GC content at the 3^rd^ position of synonymous codons (GC_3_) was determined for the complete coding sequences of the type II AFPs and the partial coding sequences of the herring and rainbow Prp8p genes using the program codonw [26].

### Phylogenetic analyses using 16S rRNA

We assembled a 16S dataset for a subset of taxa that included an outgroup (bowfin), four species with the AFP (rainbow and Japanese smelt, Atlantic herring, sea raven), and six other species mentioned above which do not possess the AFP gene according to our Southern blot and on-line searches (zebrafish, rainbow trout, Atlantic cod, winter flounder, *Takifugu rubripes* and yellow perch). Since a 16S sequence is not yet available for cisco, one of four identical sequences from four species of the same genus (*Coregonus peled*, DQ399871) was used.

The data were subjected to two analyses to test the admittedly unlikely proposition that smelts, herring and sea raven all possess type II AFPs because they form a monophyletic assemblage. First, a Bayesian analysis was conducted using the GTR+I+G model, as above, selected by MrModeltest [23]. Two independent analyses were run with Metropolis-coupled MCMC using four incrementally heated Markov chains for 1,000,000 generations until the standard deviation of the split frequencies was <0.01. Trees were sampled every 1000 generations, with the first 200 of these discarded as burn-in. A constraint tree was created in MacClade Version 4 [27] with the species possessing the AFP gene constrained to be monophyletic, and then filtered the trees resulting from our Bayesian analysis using PAUP* Version 4.0b10 [21], retaining only those that were consistent with the constraint. Our second approach employed maximum parsimony (gaps treated as missing data). We did two separate exhaustive searches for the most parsimonious tree(s) using PAUP*, one subject to our constraint tree, and the other unconstrained. We then compared the tree lengths for the most parsimonious tree(s) between the two runs.

### Southern blotting

Fish genomic DNAs were digested extensively with *Pvu*II (Fermentas) and 10 µg per lane was resolved on a 0.8% agarose gel. The DNA was transferred to zeta-probe membrane by alkaline capillary blotting as recommended by the manufacturer (Bio-Rad, Richmond, CA, U.S.A.). Probes were labeled using the random primers DNA labeling system (Invitrogen) and consisted of a portion of the rainbow smelt AFP gene (encompassing exons 3 to 6, bases 1023 to 1940 of GenBank #DQ004949), or a portion of a chicken β-tubulin cDNA (from bases 326 to 1423 of GenBank #V00389). Standard blotting techniques were used [28] except that the concentration of Denhardt's was increased to 10×, SDS to 2% and 200 µg/mL sheared and denatured calf thymus DNA was used instead of salmon DNA. All incubations and washes were done at 60°C with a final wash in 0.5% SSC, 1% SDS.

## Results

### The ten-Cys lectin-like AFPs have no close matches in the database

Extensive searches of sequences from all organisms, in all relevant GenBank databases, using herring, rainbow smelt and sea raven antifreeze proteins as the queries, revealed no close matches and no ten-Cys lectin other than the type II AFPs. These databases, including the non-redundant and EST databases, contained over 3 million cDNAs from bony fishes. The near completion of the two pufferfish (*Fugu rubripes* [29] and *Tetraodon nigroviridis* [30]), medaka (*Oryzias latipes*) [31]and zebrafish (*Danio rerio*, http://www.sanger.ac.uk/Projects/D_rerio/) genome sequences provided an opportunity to more thoroughly examine four fish species for a possible progenitor. Again, no close homologs were identified. The highest amino acid sequence identity of mature type II AFP with fish lectin-like proteins is less than 40%. The highest identity with lectins of other vertebrates and invertebrates is 33%. These values are radically different from the 85% identity between the herring and rainbow smelt AFPs.

### The high conservation of the type II AFP sequences belies their scattered distribution in fish phylogeny and is consistent with lateral gene transfer (LGT)

**Figure 3:**
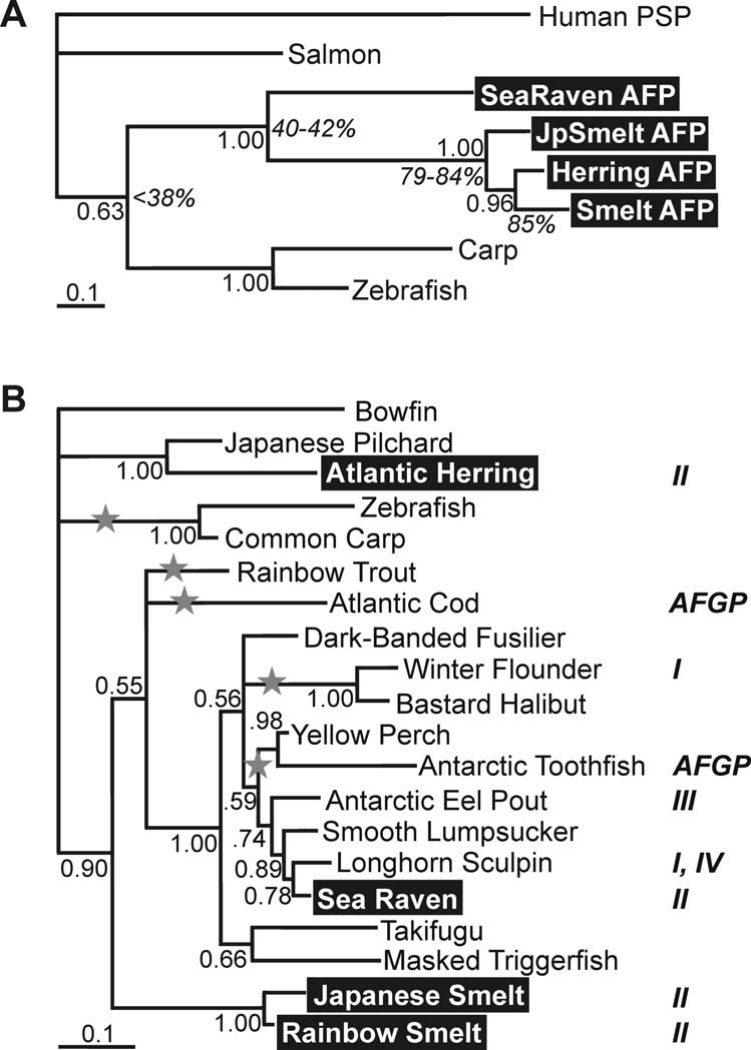
Phylogenetic trees of AFPs and related lectins as well as selected teleost fishes. (*A*) Bayesian 50% majority rule consensus tree (from 7500 sampled trees) based on the protein sequences aligned in Figure 2. The alignment was truncated to the beginning of the first helix (α0) to the end of the sea raven sequence. Branch lengths represent the expected fraction of changes, and Bayesian posterior probabilities above 50% and selected percent identities (italics) are indicated near the nodes. The highest identity between the AFPs and any other C-type lectin domain is 38%. Herring AFP is 84% and 85% identical to Japanese and rainbow smelt AFPs, respectively. The two smelt sequences share 79% identity. (*b*) Phylogenetic tree of teleosts based on ribosomal 16S RNA sequence comparison. Species are referred to by their common names. Those that are known to produce AFPs have the type of AFP they produce indicated alongside (AFGP, antifreeze glycoprotein; I, alanine-rich alpha-helical; II, lectin-like; III, beta-clip fold; IV, helix bundle). Those that produce type II AFP are highlighted. The rainbow smelt and herring sequences were double-checked by amplification from the DNAs used in this study and were almost identical to the sequences reported in the database. The non-teleost fish, the bowfin, was used to root the tree and only posterior probabilities >50% are shown. Putative type II gene loses (presuming the presence of the gene in the common ancestor) are indicated by grey stars. A further gene loss on the *Takifugu*/masked triggerfish lineage is not postulated since these fish group with yellow perch and Antarctic toothfish according to the phylogeny of Miya et al. [22].

To illustrate the discrepancy between the relatedness of the type II AFP sequences and the relatedness of the fish that produce them, Bayesian and parsimony trees were derived from the protein sequences shown in Figure 2. Since both trees were identical, only the former is presented (Figure 3A). The clustering of the type II AFP producing species (Figure 3A) based solely on the AFP sequences is completely at odds with the phylogenetic tree of teleosts based on ribosomal 16S RNA sequence comparison (Figure 3B). In contrast, the phylogenetic tree in Figure 3B is very similar to those derived from both morphology [32] and complete mitochondrial genome sequences [22]. The high similarity between the herring and rainbow smelt AFPs is amazing given that these fish diverged over 100 million years ago. The Japanese smelt confounds the already remarkable antifreeze sequence similarity, in that its AFP amino acid sequence is about as similar (84%) to that of the Atlantic herring (different superorder) as it is to the rainbow smelt sequence (same family, Figure 3).

The theoretical possibilities that herring and rainbow smelt are much more closely related than previously thought, or that the specimens were misidentified on collection, are negated by our phylogenetic tree using 16S rRNA sequences amplified from the individual Atlantic herring and rainbow smelt used in this study (Figure 3B). The herring clusters with the Japanese pilchard (same subfamily) and the rainbow smelt clusters with the Japanese smelt (same family) as expected. We also incorporated additional AFP-producing species along with their closest relatives from the tree generated by Miya et al. [22] while excluding others, to illustrate the unusual distribution of AFP types in general. Our phylogeny, generated using much less sequence data, is very similar to that of Miya et al. [22]. The two minor exceptions are at trichotomies where trout should be clustered with smelt and winter flounder/halibut should diverge earlier than takifugu/triggerfish.

If, as Liu et al. [14] have postulated, type II AFP existed in the common progenitor to the type II producing species, according to the phylogeny of Miya et al. [22], the gene must have been independently lost on at least five occasions. These theoretical losses are indicated on Figure 3B by grey stars. The absence of the gene in intervening species is supported by the database searches above and Southern blotting below.

### Similar rates of silent and missense mutations in type II AFP genes are inconsistent with strong selection for over 100 million years

Another argument against the normal descent/gene loss hypothesis, given the equivalent similarities between the AFPs of the closely related rainbow and Japanese smelts and the distantly related herring, and the greater divergence of the sea raven AFP, is that one would need to postulate starkly contrasting selection pressures on the different fishes at various times. For example, selection must have been much stronger on the herring and smelt sequences than on the sea raven sequence, but only up until the point at which the two smelts diverged. If selection was strong, the estimated ratio of non-synonymous (missense) mutations per non-synonymous site to synonymous (silent) mutation per synonymous site (*d_N_*/*d_S_*) [33] should be much less than one. However, this is clearly not the case as indicated in Table S1, since ratios close to unity are observed in all pairwise AFP comparisons. This is in stark contrast to the values obtained using the highly conserved spliceosomal protein, Prp8p [34] in which *d_N_*/*d_S_* is below 0.02 in all comparisons between herring, rainbow smelt, takifugu and zebrafish sequences. It should be noted a *d_N_*/*d_S_* ratio close to one does not imply a lack of selection for the retention of antifreeze activity in these fishes. Rather, it implies that the majority of the sites within the protein can tolerate substitutions without significantly affecting AFP function. High *d_N_*/*d_S_* ratios (averaging 0.67 and 1.0) have also been observed in the half of the residues (those not involved in ice-binding or structural integrity) of the more structurally-constrained AFP isoforms of two beetle species [35].

Another discrepancy is the differences in the proportion of silent sites that are altered. Fewer than 10% of the synonymous sites differ between the AFP sequences of herring and rainbow smelt. For Prp8p, this value increases to almost 50%, whereas for nonsynonymous sites, the opposite trend (8% for AFP vs 1% for Prp8p) is observed. A low synonymous mutation rate could be the result of selection for particular codons, which has been correlated with both GC content at the 3^rd^ position of synonymous codons (GC_3_) and expression levels in cyprinid fishes including the common carp [36]. A measure of the variability in codon usage is given by the effective number of codons (EN_c_), which ranges from 20 for genes which use but a single codon for each amino acid to 61 for genes in which codon usage is random [26]. For type II AFPs, codon usage appears quite random (Table S2) with EN_c_ and GC_3_ (brackets) values of 60 (38%) for herring, 55 (38%) for rainbow smelt, 59 (39%) for Japanese smelt and 55 (44%) for sea raven. This suggests that codon usage is close to random indicating little or no selection at silent sites. In contrast, the EN_c_ and GC_3_ values for the Prp8p genes of herring and rainbow smelt are 42 (77%) and 39 (82%) respectively, likely indicative of selection for increased GC content.

Finally, we tested the null hypothesis that the type II AFP gene is the result of normal descent in the absence of gene loss or lateral transfer, by presuming the type II AFP producing species are monophyletic. None of the 800 16S rRNA Bayesian trees was retained after filtering and the five most parsimonious constraint trees were 26 steps longer than the single most parsimonious tree without any constraint (total tree length 524 steps) meaning that monophyly of the type II producing fishes is extremely unlikely, as expected.

Taken together, the discrepancies between the 16S rRNA phylogeny and the conservation pattern of the AFPs, the high ratio (0.9) of the rate of missense to silent mutations, which suggests that the amino acid sequences of herring and rainbow smelt AFPs are not under strong selection pressure, and the low rate of silent substitution in the absence of an appreciable codon bias, are totally inconsistent with normal descent of the type II AFP gene from a common ancestor over 100 million years ago. This contrasts with the Prp8p gene, which shows a much lower rate of missense to silent mutations along with a five-fold higher rate of silent substitution with selection. An alternate and more plausible explanation for these data is that the type II AFP gene was laterally transferred into or between the herring and smelt lineages not long before the divergence of the two smelt species. LGT probably occurred on at least two occasions: in an earlier event to the ancestor of the sea raven, and more recently, to or between the herring and smelts.

### The conservation of non-coding sequences also supports LGT

**Figure 4:**
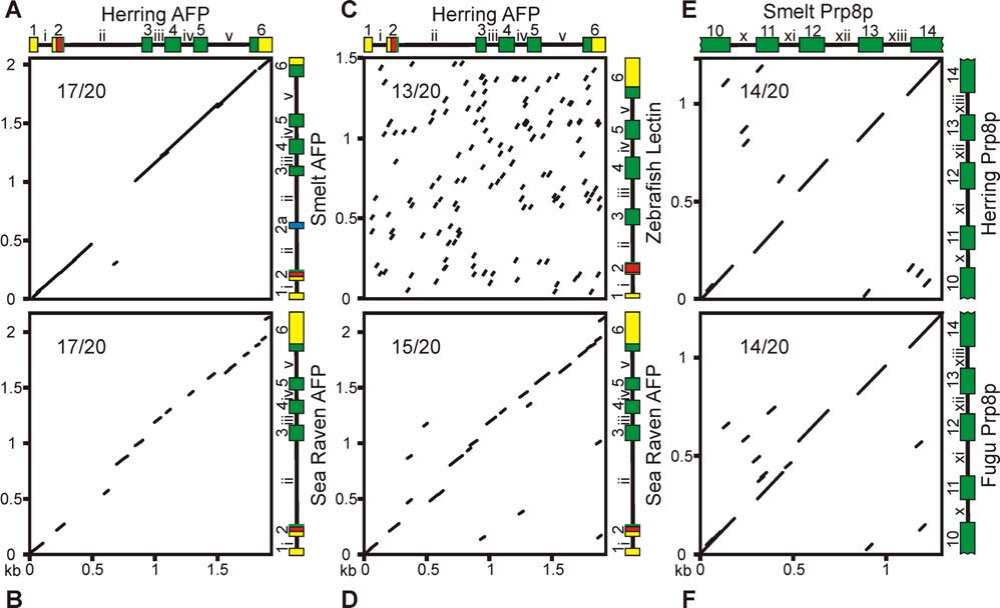
Dot matrix comparisons of lectin-like AFP and control genes. (A,B) Atlantic herring type II AFP gene (Herring, GenBank #DQ003023) compared to (A), the rainbow smelt type II AFP gene (Smelt, GenBank #DQ004949), and (B), the sea raven type II AFP gene. The genes are draw to scale with exons (boxes) numbered 1–6 and colour-coded, where yellow represents untranslated regions, red the signal polypeptide, green other coding regions, and blue the extra exon in the rainbow smelt AFP gene. Introns are designated i to v. Data points represent a 17/20 nucleotide match. (C,D) Atlantic herring type II AFP gene compared to (C), zebrafish lectin gene at a 13/20 nucleotide match, and to (D), the sea raven type II AFP gene at a 15/20 nucleotide match. (E,F) Dot matrix comparison of a segment (exons 10–14) of the rainbow smelt Prp8p gene compared to (E), the Atlantic herring Prp8p gene at a 14/20 nucleotide match and to (F), the pufferfish (*Fugu*) Prp8p gene at a 14/20 nucleotide match.

To further test the LGT hypothesis, we cloned and sequenced the introns and exons of both Atlantic herring and rainbow smelt AFPs and aligned these with the previously known sea raven sequence (Figure S1). All three genes have five introns in identical positions (Figure 4A,B). The second intron in the rainbow smelt AFP gene is interrupted by a mini-exon that codes for an N-terminal extension to the mature protein. But, this exon might be of very recent origin because its sequence is not present in the closely related Japanese smelt (*Hypomesus nipponensis*) [37]. BLAST searches, using both isolated exons and complete cDNA sequences, detected only two matches (55/65 and 50/59) with an expect value less than 10^−3^. Both correspond to sequences encoding low-complexity signal peptides, so their significance is doubtful. This paucity of sequences related to the AFP gene suggests close homologues or recognizable pseudogenes are absent from all fish and non-fish genomes sequenced to date.

Consistent with this LGT hypothesis, the AFP gene introns reveal a remarkable degree of identity of up to 97% between rainbow smelt and herring (Figure 4A; Table 1). In the dot matrix analysis, where 17 out of 20 (17/20) bases were matched for each data point, the only significant break in the alignment occurs in intron 2. Elsewhere, intron and exon sequences are equally well conserved. Although conservation of branching points and regulatory elements could account for some limited conservation between introns, this degree of intron sequence identity in fishes belonging to different superorders is unusual. Nucleotide and translated BLAST searches, using the entire gene sequence and each individual intron, only detected one additional match with an expect value less than 10^−3^. This match, of 60 out of 78 positions with two gaps, is between an uncharacterized zebrafish genomic sequence and intron 2 from herring. We do not consider this significant because it only covers 12% of the intron, there are no other matches within this contig, and this portion of the intron is not conserved between herring and rainbow smelt. As well, the only exons predicted using the gene prediction program GENSCAN [38] corresponded to the AFPs. Taken together, this suggests that these introns are unlikely to contain functional or regulatory domains unless they are specific to the AFP genes themselves.

**Table 1:** Percent identities between each intron and exon in the herring (H), rainbow smelt (S) and sea raven (SR) AFP gene sequences.

Identities – Pairwise Exclusion of Gaps – 100% = identical									
Region	SR/H % Identity		SR/H (bp)	SR/S % Identity		SR/S (bp)	H/S % Identity		H/S (bp)
	No Gaps	Gaps		No Gaps	Gaps		No Gaps	Gaps	
Exon 1	73.3	64.7	60/68	78.8	71.9	52/57	90.7	86.0	54/57
Intron 1	71.3	52.8	94/127	71.4	47.2	84/127	97.1	88.7	105/115
Exon 2	78.0	67.6	91/105	75.1	62.9	88/105	87.5	84.6	88/91
Exon 3	58.8	34.3	80/137	61.3	35.8	80/137	98.8	98.8	80/80
Intron 3	74.5	67.0	98/111	78.6	69.4	98/111	96.1	94.2	102/104
Exon 4	64.1	59.5	117/126	65.8	61.1	117/126	88.1	88.1	126/126
Intron 4	72.8	64.4	92/104	75.0	66.3	92/104	97.1	97.1	104/104
Exon 5	60.6	60.6	109/109	62.4	62.4	109/109	89.9	89.9	109/109
Intron 5	84.8	56.5	230/345	83.0	58.4	230/327	96.5	90.8	317/337
Exon 6	78.2	36.4	165/354	73.0	30.7	148/352	90.8	83.1	162/177

The columns labeled with % are the identities. The first (no gaps) is calculated by pair wise exclusion of gaps and the second (gaps) is calculated over the length of the alignment, gaps included. The column labeled with bp shows the total length of each alignment (first no gaps/second with gaps). The second intron is not included as large portions of the sequence are probably not homologous.

The herring and sea raven AFP genes also share similarity throughout their length, and the dot matrix analyses with at least 15 (or even 17) matches in a 20 base window, show again that there is conservation of both intron and exon sequences (Figure 4B,D) ranging from 34–68% identity (Table 1). In contrast, the next best match to a fish lectin sequence (zebrafish) shows no pattern of alignment for a dot matrix plot even when based on a 13/20 base match (Figure 4C). As a control, the single-copy gene sequences for a well-conserved spliceosomal protein (Prp8p) showed continuous 14/20 base matches within the exon sequences in comparisons between the rainbow smelt, herring and pufferfish (*Fugu*) genes, but no matches within the introns (Figure 4E,F; Figure S2). This lack of intron sequence identity in a gene from distant species is normal, even in one coding for a highly conserved protein. It helps make the point that the remarkable intron sequence similarity between the herring and rainbow smelt AFP genes is consistent with LGT and would be hard to explain by another mechanism.

### Genomic Southern blots confirm the absence of type II AFP gene homologs in other fishes

**Figure 5:**
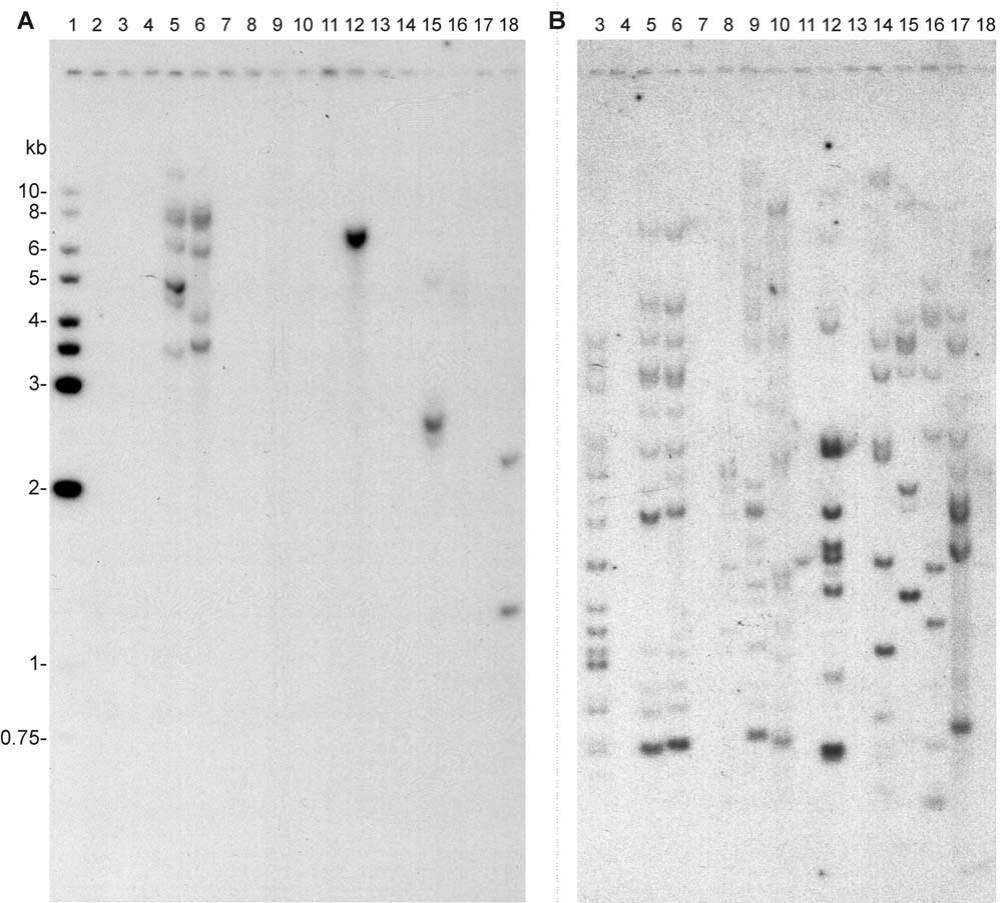
Southern blot of fish genomic DNAs. (A) Blot probed with rainbow smelt AFP genomic DNA from exon 3 to 6. (B) same blot probed with chicken beta-tubulin cDNA. Lane 1 contains markers. Lanes 2, 4, 7, 11 and 13 are blank. Other lanes contain Pvu II-digested genomic DNAs (10 µg) from the following species: 3, bowfin; 5, Atlantic herring; 6, Pacific herring; 8, zebrafish; 9, rainbow trout; 10, cisco; 12, rainbow smelt; 14, Atlantic cod; 15, sea raven; 16, yellow perch; 17, winter flounder; and 18, zebrafish. Lane 18 also contains plasmid DNAs for the herring and rainbow smelt AFP genes at a single gene copy loading.

To experimentally illustrate the sequence conservation of the type II AFP genes, and at the same time to confirm the absence of homologs in more closely related fishes, we have probed a genomic Southern blot of Pvu II-digested DNA from 11 species arrayed in order of their taxonomic relationships (Figure 5A). When the blot was probed with the 3′-half of the rainbow smelt AFP gene, encompassing both exons and introns, there was strong hybridization to a 7.8 Kb band of rainbow smelt DNA, and to multiple bands in the Atlantic and Pacific herring DNAs ranging from 3.5 to >10 Kb. There was also hybridization to the sea raven DNA at ∼2.5 Kb. However, there was no sign of hybridization to any of the other DNAs, despite the ease of detection of highly diluted control DNA at a concentration equivalent to a single gene copy. This confirms the results of the database search and illustrates that failure to find a close homolog is not due to a defect in the search strategy or a gap in the coverage of DNA sequences. When the same blot was stripped and reprobed with beta-tubulin cDNA there were signals from multiple genes in all species, illustrating that there was hybridizable DNA in each lane (Figure 5B). When the blot was reprobed with the Prp8p gene (single-copy), there were one or two hybridizing bands in each DNA-containing lane, again showing that single copy sequences can readily be detected on the blot (not shown).

## Discussion

The isolated occurrence of type II AFP in three distant branches of the teleost radiation is extraordinary. These lectin homologs are the only ones to have a fifth disulfide bridge in a specific location, and they are far more similar to each other than to any other lectin homolog. Their resemblance extends to the DNA sequence level, where even the introns are up to 97% identical. This sequence similarity is independently demonstrated by genomic Southern blotting, which also confirms the absence of type II AFP homologs in other fishes, some of which are quite closely related to the type II AFP-producing species. The most likely explanation for up to 85% amino acid sequence identity, low silent mutation rate, and extreme conservation of number, position and sequence of introns is that the type II AFP gene has been laterally transferred. Nevertheless, we have considered other possible explanations.

In the first scenario, that of gene loss, the ten-Cys type II AFP lectin homolog would have been present in the common ancestor to herring, smelt and sea raven. Since herring and smelt belong to different superorders (and in some phylogenetic schemes to different infradivisions), this ancestor would be the progenitor of nearly all teleosts. For the gene to have disappeared from those other species surveyed in the data bases and on the genomic Southern blot would require at least five gene deletion events. Taken alone, this might not be totally unexpected as it appears that notothenoid fishes that do not live in the icy Antarctic seas have lost many or all of their antifreeze glycoprotein genes [39]. But what can account for the conservation of coding sequences in the absence of strong selection as indicated by the near equivalent rates of missense and silent mutations and the low rate of silent mutations in the absense of codon selection? And how can introns that are up to 97% identical between herring and smelt after >100 million year of separation be explained?

Highly conserved sequence segments, termed ultraconserved elements, have been revealed by genome-wide comparisons between various species, including humans and fish, and some of these elements lie within introns [40]. Type II AFPs are unlikely to belong to this category of sequence, however, as most ultraconserved elements are found in the genomes of many species, whereas the distribution of type II AFP genes is extremely limited. As well, only a small proportion of introns have been shown to contain conserved noncoding elements. These can be up to several hundred base pairs in length and are thought to regulate expression of either the genes in which they lie or nearby genes [41]. Moreover, they are mainly found in and around genes that are involved in the regulation of development, which is not the function of the type II AFP gene. Although a small subset of genes may contain more than one type of ultraconserved element, these elements tend to be interspersed with regions of variable sequence, whereas the type II AFPs are highly conserved throughout most of their length. Taken together, it seems unlikely that the conservation of type II AFP exons and introns over the length of the gene can be attributed to ultraconserved elements.

Another scenario is convergent evolution of type II AFPs from lectin homologs. At least one instance of very similar AFPs appearing in divergent fishes [42] has been attributed to convergent evolution [43]. This is the occurrence of the highly repetitive antifreeze glycoproteins in Antarctic nototheniids and the unrelated Arctic cods. The former appear to have arisen *de novo* from expansion of a tripeptide sequence within the trypsinogen gene [44], [45]. A different example of convergent AFP evolution rests with the insect AFPs, where moth and beetle AFPs [46], [47] have ended up with nearly identical ice-binding sites consisting of two parallel ranks of equally spaced threonines despite being derived from very different beta-helical folds, one left-handed and the other right-handed [48]. Although one could imagine the 5^th^ disulfide bridge having been independently evolved on three separate occasions, especially if it had some functional role in ice binding, there is no way that convergent evolution could account for the overall amino acid sequence similarity and the similarity in both the third codon position and intron sequences.

Although many suggested cases of LGT, particularly between bacteria and higher eukaryotes, have been discounted [49] there is more robust evidence for LGT of mitochondrial DNA in plants [50]. Certainly, LGT between bacteria is well established and occurs frequently when there is selective pressure, as for example in the acquisition of antibiotic resistance [51]. Kurland *et al.* [52] have emphasized two criteria that bear on the success of LGT. One is that the alien sequences should not spoil the efficiency of an integrated system that has co-evolved to be optimal in that organism. The other is that for alien sequences to be perpetuated in the genome they must be adaptive. Both of these criteria are met here because 1) the antifreeze protein is presumably a single gene trait that is additional to, and largely independent of, existing systems, and 2) it is clearly of adaptive value. We suggest that the considerable selective pressure for survival in icy seawater in the face of past climate change [53], [54] has revealed the lateral transfer between fish species of a nuclear gene for freeze resistance. Indeed, the massive gene amplification that has accompanied the acquisition of AFP genes [17] is indicative of the intense selective pressure to produce adequate amounts of AFP to survive in icy seawater resulting from the Cenozoic glaciations of the last 10–20 million years [55]. Species acquiring antifreeze genes would not only have had resistance to freezing during glacial episodes but they would have faced less competition with, and predation from, non-resistant species.

There are a number of possible mechanisms that could explain LGT between species of fish, such as transfer by shared parasites, viruses or transposable elements. However, a much simpler scenario is possible. Sperm-mediated LGT is based on the ability of sperm to absorb foreign DNA from solution, and partial uptake of DNA by the sperm nucleus has been observed for many species, including zebrafish [56]. Transgenic offspring have been generated in this manner for a variety of species ranging from bees and sea urchins to fish, birds, mammals and other vertebrates (reviewed in Smith and Spadafora [57]). The exogenous DNA usually persists extrachromosomally for some time, but chromosomal integration has been observed in certain cases, such as with the fish, *Labeo rohita* [58].

Naturally-occurring sperm-mediated LGT has not yet been documented but is much more feasible for vertebrates with external fertilization, such as fish, for several reasons. During active spawning, particularly in the case of herring, the water over a huge area is often visibly discolored due to the massive release of sperm [59]. Lysis of sperm is observed in seawater [60], releasing large amounts of DNA into the water column. Although DNAses are abundant in seawater, extracellular DNA still has a half-life of several hours [61]. Also, fish eggs have a hole in their chorion (micropyle) through which the sperm and any attached DNA can enter the egg. Therefore, it is feasible that foreign DNA could be taken up by fish eggs naturally, but in most cases, it would not be retained due to failure to meet the criteria of Kurland *et al.* [52] mentioned above. However, because an AFPs gene has the potential to independently confer a strong selective advantage, it could become established in the population.

### Supporting Material

For further details see Figures S1 and S2 and Tables S1 and S2, which are available online at the XXX Web site.

## Supporting Information

Figure S1 Alignment of type II antifreeze protein gene sequences from fishes, sea raven, Atlantic herring and rainbow smelt.
(0.04 MB DOC)

Figure S2 Alignment of Prp8p sequences from various fishes.
(0.07 MB DOC)

Table S1 Estimated numbers and rates of synonymous and non-synonymous substitutions for the type II AFPs and Prp8p coding sequences of selected fish, including Rainbow smelt (Smelt) and Japanese Smelt (JpSmelt).
(0.05 MB DOC)

Table S2 Comparison of the number of times each codon is found in the type II AFP genes and the cloned portion of the Prp8p genes.
(0.15 MB DOC)
