# Supplementary material for: Lateral Transfer of a Lectin-Like Antifreeze Protein Gene in Fishes
Source: PLoS One. 2008 Jul 9;3(7):e2616. doi: 10.1371/journal.pone.0002616 (PMC2440524; doi:10.1371/journal.pone.0002616)
Supplement: Figure S1 — Alignment of type II antifreeze protein gene sequences from fishes, sea raven, Atlantic herring and rainbow smelt. (0.04 MB DOC) [file pone.0002616.s001.doc]

**Supplementary Figure S1**

Alignment of type II antifreeze protein gene sequences from fishes, sea raven, Atlantic herring and rainbow smelt. Herring and Smelt sequences were PCR amplified from genomic DNA using a primer within exon 1 and a primer within exon six. Intron sequences are italicized. Base identity between two or three of the sequences is indicated with black highlighting, excluding the non-homologous portions of intron 2 shown in lowercase font.

**Exon 1**

SeaRaven 1 TCAGCACATGAATGCAGAGGCAACAGGCTGACACTG-----AAACAAGAGAAGATATTTC
Herring 1 CTCTAAAGGGAAGACAGAGGCAACAGGCTGAAATTGTGCAGACAG---AGAAGATATTTT
Smelt 1 -----------GGGCAGAGACAACAGGCTGAAATTGTGCAGACAGAAGAGAATATATTTT

 **Intron 1**
SeaRaven 56 TACAGCAG*GTTTGCTCTCAGCCTC*--------------------*TTCTTCGTCCTGCCGA*Herring 58 TCCAGCAG*GTTTGCTCTCAGCCTCTTTTTTCTCATTGTCTTGTGTTCTTCATCCTGTGGA*
Smelt 50 TCCAACAG*GTTTGCTCTCAGCCTCTTTTTTCTCATTGTCTTGTGTTCT*----------*GT*

SeaRaven 96 *GCCCCACAGGCACTGTG-CTGCCCTGCTGTCTTTGTAATTCATTGCAACTCTTGTGTTTT*
Herring 118 *GCATCACAGTCACCATGGCTCCCCTGCACTGATTATTTCTCACT-------CTGTATTTT*Smelt 100 *GCACCACAGTCACCATGTCTCCCCTGCACTGATTATTTCTCACT-------CTGTATTTT*

 **Exon 2**
SeaRaven 155 *TCTCTTCTGATGCAG*GGCTATCAATCATCTTCATCGTCTGCACCATCTCTACCACGAGGA
Herring 171 *CTTGTTGAAG*----------------GTCATCAAGGTCAAAGTCATCTCCACTACCAGGA
Smelt 153 *CTTGTTGAAG*----------------GTCATCAACGTCAAAGTGATCTCCACCACCAGGA

 Start Pro Start

SeaRaven 215 TGCTGACTGTGTCTCTACTGGTTTGTGCCATGATGGCTCTGACTCAAGCT**A**ATGATGACA
Herring 215 TGCTGACTGTGTCTCTACTCGTTTGTGCCATCGTGGCTCTGACTAAGGCTGCTGACG---
Smelt 197 TGCTGGCTG---CTCTACTTGTTTGTGCCATGGTGGCTCTGACCAGGGCTGCAAATG---

 **Intron 2**
SeaRaven 275 *GTGAGTCTCAGTCTtacattctgtgtgtaggatactatactgtctgtaaatatattcaat*
Herring 272 *GTGAGTATCAGTCTGGAGAAATGCATTTTGTGTAGTTGTAAGCAACTTTATTCAAACACT*
Smelt 251 *GTGAGTATCGGTCTGAAGACATTTGTATTGTGTAGTTGTAAGCAACTTTATTCAAACACT*

SeaRaven 335 *tgtagacctattaagatgctgtgaatattaatattaggtaatatttagtttatttatata*Herring 332 *GAAACCA--TATATTGTACATATTATTAAATGCACGAATACGTAATGTGCTGACAAACAT*
Smelt 311 *GAAACCATTTATATTGTACATATTATTAAATGCACGAATACATAATGTGCTGACAAACAT*


SeaRaven 395 *tgtatatatatttgacagtaataa*------------------------------------
Herring 390 *GTAATTTAACGCAACAAAAACCAAGGTAATCAATTCTGCATTACAAAGACAAAACACACT*
Smelt 371 *GTAATTTAACGCAACAAAAACCAGGGTAATCAATTCTGCATTACAAAGACAAAACACACT*


SeaRaven 419 ---------------*caaaaaactaggatAGATTGCAATCCGACTTTTTGTTATCTTTAT*Herring 450 *GAACTAGAAATTTGTTCAAAATTGAGTTAAGATTTGAATATGACTTTTTTGCCTCTGTTT*Smelt 431 *AAACTAGAAATGTGTTCAAAATTGAacacatttcaattttgaacgggaaaataaatggcc*


SeaRaven 464 *TGTTAACAATATTAAAG-ACATAATTCCATAGAATTATATAATTTACATAGAAACAGCAA*Herring 510 *T----ACAATATAAAAATATATTATGCCATATAATTCTATAATTTCAGTGGAAATAGCAT*Smelt 491 *aaaagaaaaaggcactcttttaaatatgcacaacatgtttttttggcttaaaaaatttaa*

 **Smelt Exon 2a**SeaRaven 523 *ATACAACTGTCAGAGAAAG---------ACTTGACAGCTAAAGCAGGAGAGATCAAGTGT*Herring 566 *GTAAAATAGTCAAAGAGAGAGACAGACAACTTGTCAGCTAAAGCAGGAGAGATCAACTCA*
Smelt 551 *tgtgcttttcaaatgcaattgtcttatttgttctgttctgtgcatcgacag*GTGACACGG


SeaRaven 574 *agaagggagatttgatctcgtctcaactgaagctagaactgaatgtactaacttattttt*Herring 626 *ttcat-------------------------------------------------------*
Smelt 611 GGAAAGAGGCTGTGATGACAGGGTCCAGTGGAAAGAATTTGACAg*gtgcagcaacgcaac*

SeaRaven 634 *ggtgaaacaaccgaataattaattcatttttcccccacaaaactaaacgagacgcagacc*Herring 631 ------------------------------------------------------------
Smelt 671 *tgtacacagtagactatatgttaacagggttattatatggaaattagtttgttgaattga*

SeaRaven 694 *aagctaagtgtgtgctaacagtaatcagcattcgtttagcaaagtattagtaactgccat*Herring 631 *---------------------------------------ttgttacattcagttggcagt*
Smelt 731 *ttttcattattgactatagttaactgattattgttcttttacaacatttaaaagcagatg*


SeaRaven 754 *cacagcttttgactctagtggaattcatgaaatttggcagaacaaaggagacctgt----*
Herring 652 *actgtgttcatgctttgtgtagttgtaagcaagaacttcat-------------------*
Smelt 791 *cagaaacagagaaaagcaaggaaaaagaaactaagggagaattgccatattcatttttag*

SeaRaven 810 *-GCACATCTGATTCCAATGAGAATACAATGTGCTTCACAGAAAAGCACTTCAC-------*
Herring 693 *-GCACATTTAATTCCAATGAAAATACAATGTGCTTAACAGAAAAGCAAAACACAACAAAA*Smelt 851 *atactatattagatttaattttgttaacagtcttcgatagcactgcatgacactttatta*


SeaRaven 862 *CAATCCTGTACACATTCATAAAGCCACAGAAAAAAAGAGAGCTGATTAATCGTCGTTCCC*Herring 752 *CAATCCTGTACACATTCATATAGGCACAACAAATG-------TGATTAATTGTGATTCAC*
Smelt 911 *ttattatatagcctatttgtattaagcagctgtacacaggtggcgggccggtctgcgttt*

SeaRaven 922 *TCTGCTCTGA-------CAATAAAAGGATTATAAACTCCAGATTTCTGATAAACAGACTC*Herring 805 *ACCTCTTTCAGTTAAACAAATGAAAGGATTAAACACTCTT*--------------------
Smelt 971 *caaagtcctgggctgttttttggtcccagtccgaccctg*---------------------

 **Exon 3**
SeaRaven 975 *GGTGGCTTACCTGTGATCAGACATGTTACCCACTCTTC*--*TGTTTGTCCTCAG*AAATACT
Herring 845 ---*GCCCTTCCTGCGATCAGACATGTTACTCATTCTTCTCTGTTTGTCCTCAG*-------
Smelt 1010 ----------*CTGCGATCAGACATGTTACCCATTCTTCTCTCTTTGTCCTCAG*-------

Mature start
SeaRaven 1033 CAAAGGCACGGCTACAGAGGCTGGACCGGTCTCT**C**AGAGAGCCGGACCAAACTGTCCCGC
Herring 895 --------------------------------------------------AATGTCCCAC
Smelt 1053 --------------------------------------------------AATGTCCCAC


SeaRaven 1093 TGGTTGGCAACCTCTTGGTGACCGCTGTATCTATTATGAGACAACAGCGATGACTTGGGC
Herring 905 TGATTGGAAGATGTTCAATGGTCGCTGTTTCCTTTTTAATCCATTACAATTGCATTGGGC
Smelt 1063 TGATTGGAAGATGTTCAATGGTCGCTGTTTCCTTTTTAATCCATTACAATTGCATTGGGC

 **Intron 3**
SeaRaven 1153 TCTGGCTGAG*GTAGTCAGGATATGATTATGATTCAGATTGCTTCTAAACTGGTCTGGTGG*
Herring 965 TGACGCTCAG*GTAATCAGAATG*-------*GATTCAGATTGATTTGAAACTGTTCTGGTGG*
Smelt 1123 TCACGCTCAG*GTAATCAGAATG*-------*GATTCAGATTGCTTTTAAACTGTTCTGGTGG*


SeaRaven 1213 *TATTGCCTTACATGCTCGGTTAATTGAGCATGAGCTT*------*GACTCATTTCCACTGCA*
Herring 1018 *TATTGACTTGCATGCTCGGTTATTTGACATCTTCGTCTTAC--TGCACATCTCCACTGTA*
Smelt 1176 *TATTGACTTGCATGCTCGGTTATTTGACATCTTCGTCTGACACGGCACATCTCCACTGTA*
 **Exon 4**
SeaRaven 1267 *G*ACAAACTGTATGAAATTGGGTGGACACCTTGCATCCATCCACAGCCAGGAGGAGCATAG
Herring 1076 *G*GAAAGCTGTATGAAGGAGGGGGCAAACCTTGCATCCATTCACAGCCTTGAAGAGTCTAC
Smelt 1236 *G*ATAAGCTGCATGAAGGATGGGGCAAACCTTGCATCCATACACAGCCTTGAAGAGTATGC

SeaRaven 1327 TTTCATTCAGACCTTGAATGCTGGTGTTGTA---------TGGATCGGAGGCTCCGCTTG
Herring 1136 GTTTGTTAAGGAGCTGACAAGTGCAGACTTAATCCCATCATGGATTGGAGGCACAGATTG
Smelt 1296 GTTTGTTAAGGAGCTGACAACTGCAGGCTTAATACCAGCATGGATTGGAGGCTCAGATTG

 **Intron 4**
SeaRaven 1378 CCTCCAG*GTAAAA*--------*CATTGCATTACAATGG---TGGCAGAAAGA-AAGGATTT*
Herring 1196 CCAAATA*GTAATTTTTCACTACACAATGTTTTGATGGTACTGATAGAATGAGAATGATTT*
Smelt 1356 CCATGTG*GTAATATTTCACTACACAAGGTTTTGATGGTACTGATAGAATGAGAATGATTT*

 **Exon 5**
SeaRaven 1426 *TTATTACATGCTATCTTACTATACGTATATTCTTTCCTTTCTGTTTTCTAG*GCAGGTGCT
Herring 1256 *ATATTATACACTATTTTATTACACATATACTCTTTCTTTTCTGTTTTTTAG*TCAACCCGT
Smelt 1416 *ATATTATACACTATTTTATTATACATATACTCTTTCTTTTCTGTTTTTTAG*TCAACATAT


SeaRaven 1486 TGGACCTGGTCTGATGGTACACCTATGAATTTTCGTTCCTGGTGTTCTACCAAACCTGAT
Herring 1316 TGGTTTTGGATGGATAGCACAGGTATGGATTATGCTGACTGGTGCGCTGCACAACCTGAT
Smelt 1476 TGGTTTTGGATGGATAGTACAAGTATGGATTTTACTGACTGGTGCGCTGCACAACCTGAT

 **Intron 5**SeaRaven 1546 GATGTACTGGCCGCGTGCTGTATGCAGATGACTGCTGCAG*GTAAATCACAACACATT---*
Herring 1376 ACTACCTTAACTGAGTGCTGCATACAGATGAATGTTGGAA*GTAAGTCACAACTCATTGTT*
Smelt 1536 TTTACCTTAACTGAGTGCTGCATACAGATAAATGTTGGAG*GTAAGTCACAACTCATTATT*


SeaRaven 1603 *-------AGAGCATAGTATTAAATGACTGAAGGCAGTAGTGTTGTTTA-GTACATTT---*Herring 1436 *TTGCTTAAGAGCATACTATTAAATGACTGAAGGCAGTAGTGTTGTTTTTGTACATTTTGT*
Smelt 1596 *TTGCTTAAGAGCATACTATTAAATGACTGAAGGCAATAGTGTTGTTTCTGTGCATTTTGT*

SeaRaven 1652 ------------------------------------------------------------
Herring 1496 *AAGTTAATGGCATTTTTGTCAGTTAAAATCAG-AAGTAAATAATACACATTGCTTTGTTT*
Smelt 1656 *AA-------------------GTTAAAATCACTAAGTAAATAATACACATGGCTTTGTTT*


SeaRaven 1652 -----------------------*GGTTCATCTTGAGATCAATACTCTCAGAATTTCACTT*
Herring 1555 *AGAGTAAAATCGAATTGTCTTGAGGTTGATCTTGAGATCAATACTTTCTGCATTTCACAT*Smelt 1697 *AGAGTAAAACCGAATTGTCTTGAGGTTGATCTTGAGATCAATACTTTCTGCATTTCACAT*


SeaRaven 1689 *TTGAATCACTTTTGTTCTTCAGTTCATGTGTAGCTTTGGCCTCGTTATCCGTGTCTTTGT*
Herring 1615 *TTGAATCGCTTTGGTTCCTCAGTTCATACGTAGCTTTTGTCTT-------TTGTCTTTGT*
Smelt 1757 *TTTAATCGTTTTGGTTCCTCAGTTCATACGTAGCTTTTGTCTT-------TTGTCTTTGT*
 *SeaRaven 1749 CTGTCTAGTGATGAAG*---------*ACAGTTTCAGGTTA-GGTTGGTATGGCGCTGACTC*Herring 1668 *CTGTG-AGTAGCGATGTGAGTGTGGCCTCGTTCAGGTTTTGGTTGGTGTAGAATTGCCTG*
Smelt 1810 *CTGTG-AGTAGTGATGTGAGTGTGGCCTCGTTCAGGTTTTGGTTGGTGTAGAATTGCCTG*

**Exon 6**
SeaRaven 1799 *ACTTCTTGTGTTTTTGATGTTTACAG*CTGACCAATGCTGGGATGACTTGCCTTGTCCGGC
Herring 1727 *ACTTCT-GTGTTTTTGATGTTTACAG*TTGGAAAATGCTGGAATGACACACCTTGTACGCA
Smelt 1869 *ACTTCT-GTGTTTTTGGTGTTTACAG*TTGGAAAATGCTGGAATGACACACCTTGTACCC*A*

 Stops
SeaRaven 1859 GTCCCACAAATCAGTCTGCGCCATGACATTCTA-----------AGCTAACACAGAGGCC
Herring 1786 TCTTCATTCATCAATCTGCGCCAAGCCACTGAA-ATGATTCCTGAGCTGACACAGAGGC-
Smelt 1928 TCTTCATGCATCAGTCTGCGCCAAGCCTGCCACCGTGATTCCCGAGGTGACAC-------


 Stop

SeaRaven 1908 ATCCATCAC--ACAAACACTTTAGTGGGTGTTTGATTGTGTGT--GTTCGCATACTCATC
Herring 1844 ATCCACCACCATCAATCACG------------TGATTGTGTGTATGTTTGCATACTCTTC
Smelt 1981 ------CACCATCAATCATG------------TGATTGTGTGTATGTTTGCATACTCTTC


SeaRaven 1964 TGTGTTCGTGTCAACAGCCTCATGCTGAACCTGAAGGTTCAAAATCTCATATGACATCTT
Herring 1892 TGTGT-------------------------------------------------------
Smelt 2023 TGA---------------------------------------------------------


SeaRaven 2024 TAATTCTTTGCTATTGTTGGAGCTGCCTGAAAGGATGAGACGACAAGAGCTGGAAAGCAT
Herring 1897 ------------------------------------------------------------
Smelt 2026 ------------------------------------------------------------


SeaRaven 2084 CTGAGGGCTTTTAGGAAGAAATTGAATGGTTATGAAAATGATGGTCTTTTTATGTATTAT
Herring 1897 ----------------------------------------------TTTTCATGTATCAA
Smelt 2026 ----------------------------------------------TTTTCATGTATCAA


SeaRaven 2144 GTCAAATTAAAAGGCTGACACGTTG
Herring 1911 GTCAAAT-AAAAGGCTGAAAC----
Smelt 2040 GTCAAAT-AAAAGGCTTG-------

The herring, smelt and sea raven AFP genomic sequences can be obtained from the GenBank nucleotide database under accession numbers DQ003023, DQ004949 and J05100 respectively.
