# Supplementary material for: Lateral Transfer of a Lectin-Like Antifreeze Protein Gene in Fishes
Source: PLoS One. 2008 Jul 9;3(7):e2616. doi: 10.1371/journal.pone.0002616 (PMC2440524; doi:10.1371/journal.pone.0002616)
Supplement: Figure S2 — Alignment of Prp8p sequences from various fishes. (0.07 MB DOC) [file pone.0002616.s002.doc]

**Supporting Fig. 2**

Alignment of Prp8p sequences from various fishes. A portion of Danio and Fugu Prp8p cDNA and genomic sequences were obtained from databases. The Smelt and Herring genomic sequences were PCR amplified from genomic DNA, as described for the AFP genes, using the primers indicated in yellow highlighting.

ZebrafcDNA CAGCCTGTGAAGGTGCGTGTGTCTTACCAGAAGCTTCTGAAGTACTATGTGTTGAATGCT

FugucDNA CAGCCTGTGAAAGTGCGTGTGTCGTACCAGAAATTGCTCAAGTATTATGTGCTCAATGCT

Zebrafgene CAGCCTGTGAAGGTGCGTGTGTCTTACCAGAAGCTTCTGAAGTACTATGTGTTGAATGCT

Fugugene CAGCCTGTGAAAGTGCGTGTGTCGTACCAGAAATTGCTCAAGTATTATGTGCTCAATGCT

Smeltgene -----------------------CTACCAGAAGTTGCTGAGGTACTACGTGCTCAACGCT

Herringgene ------------------------------------------------------------

HumancDNA CAGCCTGTGAAAGTGAGGGTCTCCTACCAGAAGCTGCTTAAGTACTATGTGCTGAATGCC

ZebrafcDNA CTAAAACACAGACCACCAAAGGCACAGAAAAAGAG-------------------------

FugucDNA CTCAAGCATCGACCACCAAAGGCACAAAAGAAGAG-------------------------

Zebrafgene CTAAAACACAGACCACCAAAGGCACAGAAAAAGAGGTGAGTCTGCTATTCCAGAGCCTGT

Fugugene CTCAAGCATCGACCACCAAAGGCACAAAAGAAGAGGTAAATGTAATATAGATAAATAATG

Smeltgene CTCAAGCACAGACCACCCAAGGCCCAGAAGAAGAGGTGAGCAGCGATACATTTTTTATCA

Herringgene ------------------------------------------------------------

HumancDNA CTGAAGCATCGGCCCCCTAAGGCTCAAAAGAAGAG-------------------------

ZebrafcDNA ------------------------------------------------------------

FugucDNA ------------------------------------------------------------

Zebrafgene AGCAAGGTGTAATTTAAAAAGAGGTGTGTTGTAATGCATATAACAATGGTTGTAT-----

Fugugene GATAGCAGGATTTCAATCTTTCCTTGTGATGCAAGTGGCTTTCATAGGAAGCTAAAAATA

Smeltgene ATTATGGAAGTTGAAATATCAGATGGATTGTAGAGTGATTGACAAGACATCTGAAAATAA

Herringgene ------------------------------------------------------------

HumancDNA ------------------------------------------------------------

ZebrafcDNA -----------------------ATACCTGTTCCGCTCTTTCAAGGCCACCAAATTCTTC

FugucDNA -----------------------GTACCTCTTCCGCTCTTTCAAGGCCACCAAGTTCTTT

Zebrafgene ----TTTCCTTTCTCTTCTTTAGATACCTGTTCCGCTCTTTCAAGGCCACCAAATTCTTC

Fugugene ----TTTTCTATTTTTCTTTCAGGTACCTCTTCCGCTCTTTCAAGGCCACCAAGTTCTTT

Smeltgene TAATTTCCCCCCACGATTTCCAGGCACCTCTTCCGCTCGTTCAAGGCCACCAAGTTCTTC

Herringgene -----------------------------------------------------GTTCTTC

HumancDNA -----------------------GTATTTGTTCCGCTCCTTCAAAGCCACCAAATTCTTT

*****

ZebrafcDNA CAGTCAACAAAGCTGGACTGGGTAGAAGTCGGACTCCAGGTGTGCCGGCAGGGCTACAAC

FugucDNA CAGTCTACCAAGCTGGACTGGGTGGAAGTGGGTCTGCAGGTGTGTCGGCAGGGCTACAAC

Zebrafgene CAGTCAACAAAGCTGGACTGGGTAGAAGTCGGACTCCAGGTGTGCCGGCAGGGCTACAAC

Fugugene CAGTCTACCAAGCTGGACTGGGTGGAAGTGGGTCTGCAGGTGTGTCGGCAGGGCTACAAC

Smeltgene CAGTCAACCAAGCTGGATTGGGTGGAGGTGGGCCTGCAGGTCTGCAGACAGGGCTACAAC

Herringgene CAGTCCACTAAGCTGGACTGGGTGGAGGTCGGGCTGCAGGTGTGCAGGCAGGGCTACAAC

HumancDNA CAGTCCACAAAGCTGGACTGGGTGGAGGGTTGGCTCCAGGTTTGCCGCCAGGGCTACAAC

***** ** ******** ***** ** * * ** ***** ** * ************

ZebrafcDNA ATGCTGAACCTGCTGATTCACAGAAAGAACCTGAACTACCTGCACTTGGACTACAACTTC

FugucDNA ATGCTCAATCTGCTTATCCACCGCAAGAACCTGAACTACCTGCATCTTGACTACAACTTC

Zebrafgene ATGCTGAACCTGCTGATTCACAGAAAGAACCTGAACTACCTGCACTTGGACTACAACTTC

Fugugene ATGCTCAATCTGCTTATCCACCGCAAGAACCTGAACTACCTGCATCTTGACTACAACTTC

Smeltgene ATGCTTAACCTGCTCATCCACCGTAAGAACCTCAACTACCTGCATCTGGACTACAACTTC

Herringgene ATGCTGAACCTGCTCATTCACAGAAAGAACCTCAACTACCTGCATCTGGATTACAACTTC

HumancDNA ATGCTCAACCTTCTCATTCACCGCAAAAACCTCAACTACCTGCACCTGGACTACAACTTC

***** ** ** ** ** *** * ** ***** *********** * ** *********

ZebrafcDNA AACTTGAAGCCTGTAAAAACCCTCACAACAAAG---------------------------

FugucDNA AACCTGAAGCCTGTCAAGACACTGACCACAAAG---------------------------

Zebrafgene AACTTGAAGCCTGTAAAAACCCTCACAACAAAG---------------------------

Fugugene AACCTGAAGCCTGTCAAGACACTGACCACAAAGGTAAAGTTCTTACTGTTTAAAGTGTTT

Smeltgene AACCTGAAGCCCGTCAAGACGCTCACCACCAAGGTATGGAGCGATCCAAGTTTTTCTTTT

Herringgene AACCTCAAGCCCGTCAAGACCCTGACCACAAAGGTGAGTTGTCCACAATGAGACGAGTCG

HumancDNA AACCTCAAGCCTGTGAAAACGCTCACCACCAAG---------------------------

*** * ***** ** ** ** ** ** ** ***

ZebrafcDNA ------------------------------------------------------------

FugucDNA ------------------------------------------------------------

Zebrafgene ------------------------------------------------------------

Fugugene TTATGATGATCCTTTAAAAAAAAACTTGTTACATTTGTACAGAATTTTTCTGTAC-----

Smeltgene TAAGCCCTTATTTGTACCCTGCAGTCAGTGCTCTGTGTTGTTGTTGTCATTTGTGTGTTT

Herringgene ACGTGAGCTCAGGTTTTTCGTGTGACTGTCAGG---------------------------

HumancDNA ------------------------------------------------------------

ZebrafcDNA -----------------------------------------------------GAACGTA

FugucDNA -----------------------------------------------------GAACGAA

Zebrafgene -----------------------------------------------------GAACGTA

Fugugene -------------------GTTATTTGCCATTAATATATTTATCTAATTCTAGGAACGAA

Smeltgene GGATTACTAGCCTGGTATAATGTAGGCCTGTGTGGTTTCTTTCTCTCCTTCAGGAGCGTA

Herringgene -------------------TGTCCTCATACATGCTCGTCTTTCGCCGCCACAGGAACGTA

HumancDNA -----------------------------------------------------GAAAGAA

** * *

ZebrafcDNA AGAAGTCTAGATTCGGAAATGCGTTTCACTTGTGCCGTGAGGTTCTGCGCTTGAGCAAGC

FugucDNA AGAAATCCAGATTTGGCAATGCCTTCCACTTATGCAGAGAGGTGTTGCGTCTCAGTAAGC

Zebrafgene AGAAGTCTAGATTCGGAAATGCGTTTCACTTGTGCCGTGAGGTTCTGCGCTTGAGCAAGC

Fugugene AGAAATCCAGATTTGGCAATGCCTTCCACTTATGCAGAGAGGTGTTGCGTCTCAGTAAGC

Smeltgene AGAAGTCCAGATTCGGAAATGCCTTCCATCTGTGCAGAGAGGTGTTGCGTCTCAGCAAGC

Herringgene AGAAGTCCAGATTCGGAAACGCGTTCCACTTGTGCAGAGAGGTGCTTCGCCTCAGCAAGC

HumancDNA AGAAATCTCGTTTTGGGAATGCTTTCCACCTGTGTCGGGAAGTTCTGCGTTTGACTAAGC

**** ** * ** ** ** ** ** ** * ** * ** ** * ** * * ****

ZebrafcDNA TGGTGGTGGACAGTCATGTACAGTACAGGCTTGGAAATGTTGATGCTTTCCAG-------

FugucDNA TGGTGGTGGACAGCCATGTGCAGTACAGACTTGGAAATGTTGATGCCTTCCAG-------

Zebrafgene TGGTGGTGGACAGTCATGTACAGTACAGGCTTGGAAATGTTGATGCTTTCCAGGTATAAC

Fugugene TGGTGGTGGACAGCCATGTGCAGTACAGACTTGGAAATGTTGATGCCTTCCAGGTGTGTT

Smeltgene TAGTGGTGGACAGCCACGTGCAGTACAGACTGGGCAACGTCGACGCCTTCCAGGTGAGGG

Herringgene TGGTGGTGGACAGCCACGTGCAGTACAGGCTGGGCAATGTTGACGCTTTCCAGGTAAGGT

HumancDNA TGGTGGTGGATAGTCACGTGCAGTATCGGCTGGGCAATGTGGATGCCTTCCAG-------

* ******** ** ** ** ***** * ** ** ** ** ** ** ******

ZebrafcDNA ------------------------------------------------------------

FugucDNA ------------------------------------------------------------

Zebrafgene AAATACAACCTTTATGCACTCTGTGAAGCTTGTTAGTTTTGTGACCACTAA---------

Fugugene AAACAACAAAGCTTTGTCACAAACAGTGTGTTATTGTCAAGAAAGACTTTAAACAATGAT

Smeltgene GCACTTATTTACATACTGAACTGTACTGTTTTTACAAAGAAAGCTTCACCGTGGTATTGA

Herringgene TCTCCCGGAGATCGTATTTGTTTTAAATTAGGAGTTGTGTCGCCGAGTTCGTTTGTAAGC

HumancDNA ------------------------------------------------------------

ZebrafcDNA ------------------------------------------------------------

FugucDNA ------------------------------------------------------------

Zebrafgene ------------------------------------------------------------

Fugugene TTACTTTTCTTTTTCACTTTCGAAAATTTGAACTTGATATGTTGTCGTGGGACATTTTTA

Smeltgene GGAAGCAAATGGCTAAGGT-----------------------------------------

Herringgene CAGCAGCCAAAGGCTAGACACTTATAGCCATTTTTCAAAATGTATTTCAAACATAGTTTC

HumancDNA ------------------------------------------------------------

ZebrafcDNA ------------------------------------------------------------

FugucDNA ------------------------------------------------------------

Zebrafgene ----------------------------------------TAAACTTGTCTGCCTTCCTG

Fugugene TAGATTAACATAAATTTTG-----------ACTAATCCTCGAATGTTTTTATTTGTACTG

Smeltgene --------------------------------------ACACTGCCTCCTCCCATCTCTG

Herringgene TCACTGTTTTAAGCATGGTCATGGTTCTCGTTGCACGTACTCATTGTCGTGTCTTATCTG

HumancDNA ------------------------------------------------------------

ZebrafcDNA ---CTTGCTGATGGCTTGCAGTACATCTTTGCCCATGTGGGTCAACTGACTGGCATGTAC

FugucDNA ---TTGTCTGATGGGCTGCAATACATCTTTGCTCACGTGGGTCAGCTGACGGGCATGTAC

Zebrafgene TAGCTTGCTGATGGCTTGCAGTACATCTTTGCCCATGTGGGTCAACTGACTGGCATGTAC

Fugugene TAGTTGTCTGATGGGCTGCAATACATCTTTGCTCACGTGGGTCAGCTGACGGGCATGTAC

Smeltgene TAGCTTTCAGATGGGATTCAGTACATCTTTGCCCACGTGGGCCAGCTGACAGGCATGTAC

Herringgene CAGCTGTCTGACGGCCTGCAGTATATCTTCGCCCATGTGGGCCAGCTGACGGGCATGTAC

HumancDNA ---CTGGCAGATGGATTGCAGTATATATTTGCCCATGTTGGGCAGTTGACGGGCATGTAT

* * ** ** * ** ** ** ** ** ** ** ** ** **** ********

ZebrafcDNA CGCTACAAGTACAAGCTCATGAGGCAGATCAGAATGTGCAAAGACCTGAAGCATCTCATC

FugucDNA CGCTACAAATACAAGTTGATGAGACAAATCCGGATGTGCAAAGACCTGAAGCATCTCATC

Zebrafgene CGCTACAAGTACAAGCTCATGAGGCAGATCAGAATGTGCAAAGACCTGAAGCATCTCATC

Fugugene CGCTACAAATACAAGTTGATGAGACAAATCCGGATGTGCAAAGACCTGAAGCATCTCATC

Smeltgene CGCTACAAGTACAAGCTGATGAGACAGATCCGCATGTGCAAGGATCTCAAGCATCTCATC

Herringgene CGCTACAAGTACAAGCTGATGAGACAGATCCGCATGTGCAAGGACCTGAAGCATCTCATC

HumancDNA CGATACAAATACAAGCTGATGCGACAGATTCGCGTGTGCAAGGACCTGAAGCATCTCATC

** ***** ****** * *** * ** ** * ******* ** ** ************

ZebrafcDNA TACTATCGCTTCAACACA------------------------------------------

FugucDNA TACTACCGGTTCAACACT------------------------------------------

Zebrafgene TACTATCGCTTCAACACAGTAAGTAGCACTCAATGGGGTTATCAAAAAACATTGTTTCTG

Fugugene TACTACCGGTTCAACACTGTAAGTTAATGTTGGATTGCATCCAGCAGTTGCCCTTGCGCA

Smeltgene TACTACCGCTTCAACACTGTGAGTACACGTACACACACTTCATTTGTGTTCATGTAAAAG

Herringgene TACTACCGCTTCAACACCGTGAGTACAGGCCTCAGGGTTATGACCTGCACAGATGTTGAG

HumancDNA TATTATCGTTTCAACACA------------------------------------------

** ** ** ********

ZebrafcDNA ------------------------------------------------------------

FugucDNA ------------------------------------------------------------

Zebrafgene TCACATGTGATGGATGGATGGTTTGGATTG------------------------------

Fugugene GGGATTTTCTGTCCTTTGCCCA--------------------------------------

Smeltgene ATCTAGCGTATTCACAAGGCATTGTAGCCAAGGTACACAAGTACTACTTGTGTGAAACTA

Herringgene GCGTACTGACCTGTTCAAATAGGGAAGAGT------------------------------

HumancDNA ------------------------------------------------------------

ZebrafcDNA ------------------------------------------------------------

FugucDNA ------------------------------------------------------------

Zebrafgene -------------------------------------------ATGCTATTCCTTGCAAC

Fugugene -------------------------------------------TATATTTATTTTTTTAT

Smeltgene ACATTTTTATCCTAACCGCCACCATAACCATGGCAATTACTACGCCCACCCTCATGTTGG

Herringgene -------------------------------------CTGGTAAACCCTGTGTGAAGTGT

HumancDNA ------------------------------------------------------------

ZebrafcDNA --------------------GGCCCAGTTGGAAAAGGTCCAGGCTGTGGGTTCTGGGCAC

FugucDNA --------------------GGTCCTGTGGGCAAGGGTCCAGGTTGTGGCTTCTGGGCTC

Zebrafgene TAAAGTCATCTATCTTTTAGGGCCCAGTTGGAAAAGGTCCAGGCTGTGGGTTCTGGGCAC

Fugugene CTTTATCTTGTTTGCGTCAGGGTCCTGTGGGCAAGGGTCCAGGTTGTGGCTTCTGGGCTC

Smeltgene GTGTCATTGTATGTTCCCAGGGCCCTGTGGGCAAGGGTCCAGGCTGTGGCTTCTGGGCAC

Herringgene CGTGATGTGTTTGCTTGTAGGGACCGGTGGGCAAAGGCCCTGGCTGTGGATTCTGGGCTC

HumancDNA --------------------GGCCCTGTAGGGAAGGGTCCTGGCTGTGGCTTCTGGGCTG

** ** ** ** ** ** ** ** ***** ********

ZebrafcDNA CAGGATGGAGAGTCTGGCTGTTCTTCATGAGAGGCATTACCCCTCTTTTGGAGCGGTGGC

FugucDNA CTGGCTGGAGAGTGTGGCTATTCTTTATGAGGGGGATCACTCCTCTGTTAGAGAGATGGC

Zebrafgene CAGGATGGAGAGTCTGGCTGTTCTTCATGAGAGGCATTACCCCTCTTTTGGAGCGGTGGC

Fugugene CTGGCTGGAGAGTGTGGCTATTCTTTATGAGGGGGATCACTCCTCTGTTAGAGAGATGGC

Smeltgene CGGGCTGGAGGGTGTGGCTCTTCTTCATGAGGGGCATCACCCCTTTGCTGGAAAGATGGT

Herringgene CTGGGTGGCGTGTCTGGTTGTTCTTCATGAGGGGTATAACGCCACTGCTGGAGAGATGGC

HumancDNA CCGGTTGGCGAGTCTGGCTCTTTTTCATGCGTGGCATTACCCCTTTATTAGAGCGATGGC

* ** *** * ** *** * ** ** *** * ** ** ** ** * * ** * ***

ZebrafcDNA TCGGCAATCTTCTGGCTAGGCAGTTTGAGG------------------------------

FugucDNA TGGGAAATCTGCTGGCCAGGCAGTTTGAAG------------------------------

Zebrafgene TCGGCAATCTTCTGGCTAGGCAGTTTGAGGGTAAAATAAGCTTTGTATTTACAAAGGATG

Fugugene TGGGAAATCTGCTGGCCAGGCAGTTTGAAGGTATGTGTAGGCTTTTTTGTTAAAGGATGT

Smeltgene TGGGCAACCTGCTGGCCAGGCAGTTTGAAGGTATGACCCCTTTTTTCACCTGACTTGAGT

Herringgene TTGGAAACCTGCTGGCCAGGCAGTTTGAGGGTAACTATCACATGTTTACTTCAATAAGTG

HumancDNA TTGGCAACCTCCTGGCCCGGCAGTTTGAAG------------------------------

* ** ** ** ***** ********** *

ZebrafcDNA ------------------------------------------------------------

FugucDNA ------------------------------------------------------------

Zebrafgene CTGAAAGGTGGTGTTCTGGATTA-------------------------------------

Fugugene TTTAGCAGAAATATTTTTTGCTATAAATAAATAGAACCTAAAG-----------------

Smeltgene AAGATACTTGAGAATAGGAACTTTAATCTGTAACAGAATTTATTTTATGACATCTGAACT

Herringgene GAGATATTTATCATTGGTATTACTGTATCTTAAGTGTAGGAGTGGAGA------------

HumancDNA ------------------------------------------------------------

ZebrafcDNA ------------------------------------------------------------

FugucDNA ------------------------------------------------------------

Zebrafgene -------------------------------------ATTTGATGTGTATTGCTGTTTAG

Fugugene -----------------------TTTAAGCAATGCTACCCCATTTCAATTACTCCCGCAG

Smeltgene CATAAGCAGTAATATGATCGGTCATTTGACTACAACCCCCATATTCCTGCCTGTAAACAG

Herringgene -----------------------TACGACCGGTTGACTGACGTTTTGATGTGTCTTCTAG

HumancDNA ------------------------------------------------------------

ZebrafcDNA GTCGACACTCTAAGGGTGTTGCCAAGACTGTGACCAAACAGCGTGTGGAATCTCACTTTG

FugucDNA GACGTCATTCCAAGGGTGTAGCCAAGACGGTTACGAAACAGCGTGTGGAGTCTCACTTTG

Zebrafgene GTCGACACTCTAAGGGTGTTGCCAAGACTGTGACCAAACAGCGTGTGGAATCTCACTTTG

Fugugene GACGTCATTCCAAGGGTGTAGCCAAGACGGTTACGAAACAGCGTGTGGAGTCTCACTTTG

Smeltgene GCCGTCACTCCAAGGGTGTGGCCAAGACTGTGACTAAGCAGCGAGTGGAGTCCCACTTCG

Herringgene GCCGACACTCCAAGGGAGTTGCGAAGACAGTCACCAAGCAGCGTGTGGAGTCCCACTTTG

HumancDNA GTCGACACTCAAAGGGGGTGGCAAAGACAGTAACAAAGCAGCGAGTGGAGTCACATTTTG

* ** ** ** ***** ** ** ***** ** ** ** ***** ***** ** ** ** *

ZebrafcDNA ATCTGGAGCTGAGAGCTGCTGTGATGCATGACATCCTGGATATGATGCCTGAGGGTATCA

FugucDNA ACCTGGAGTTGCGTGCTGCTGTGATGCACGATATCTTGGACATGATGCCTGAGGGTATCA

Zebrafgene ATCTGGAGCTGAGAGCTGCTGTGATGCATGACATCCTGGATATGATGCCTGAGGGTATCA

Fugugene ACCTGGAGTTGCGTGCTGCTGTGATGCACGATATCTTGGACATGATGCCTGAGGGTATCA

Smeltgene ACCTGGAGCTGCGTGCTGCCGTCATGCACGACATCCTGGACATGATGCCTGAGGGGATCA

Herringgene ACCTGGAGCTCCGTGCTGCCGTGATGCACGACATTCTAGACATGATGCCCGAGGGGATCA

HumancDNA ACCTTGAGCTGCGGGCAGCTGTGATGCATGATATTCTGGACATGATGCCTGAGGGGATCA

* ** *** * * ** ** ** ***** ** ** * ** ******** ***** ****

ZebrafcDNA AGCAAAACAAGGCCAGAACCATCTTGCAGCACCTCAGTGAGTCATGGAGATGCTGGAAGG

FugucDNA AACAAAACAAGGCCAGAACCATCTTGCAGCACCTCAGCGAGTCTTGGAGATGCTGGAAGG

Zebrafgene AGCAAAACAAGGCCAGAACCATCTTGCAGCACCTCAGTGAGTCATGGAGATGCTGGAAGG

Fugugene AACAAAACAAGGCCAGAACCATCTTGCAGCACCTCAGCGAGTCTTGGAGATGCTGGAAGG

Smeltgene AGCAGAACAAGGCCAGGACCATCCTGCAGCACCTCAGTGAGTCCTGGAGATGC-------

Herringgene AGCAGAACAAGGCCAGAACCATCCTGCAGCACCTCAGCGAGTCCTGGAGATGCTGGAAAG

HumancDNA AGCAGAACAAGGCCCGGACAATCCTGCAGCACCTCAGTGAAGCCTGGCGCTGCTGGAAAG

* ** ********* * ** *** ************* ** * *** * ***

ZebrafcDNA CCAACATTCCTTGGAAG-------------------------------------------

FugucDNA CCAACATTCCTTGGAAG-------------------------------------------

Zebrafgene CCAACATTCCTTGGAAGGTACAGTTTTGTTGCTTATAAATATTATTCAGATTTAAAAGTT

Fugugene CCAACATTCCTTGGAAGGTCTGTGTCTCATGGAGTGTAATTACTGTTAAACCTAGGTTTT

Smeltgene ------------------------------------------------------------

Herringgene CCAACATTCCATGGAAGGTTAGGCTTGTTGAAATATCCAGTGTTTCACCCGTGGCAAATA

HumancDNA CCAACATTCCCTGGAAG-------------------------------------------

ZebrafcDNA ------------------------------------------------------------

FugucDNA ------------------------------------------------------------

Zebrafgene TATCA-------------------------------------------------------

Fugugene TAAGGAAGGTGTTCAAGGTTTACAGTATTCACAGGCTGTTTAAGCTTTGTAGTTATTGTG

Smeltgene ------------------------------------------------------------

Herringgene AATCATTTGTATTAGGAAGTATTTTGGAATTTTCTCACAGAATATGAAGATTGTTATCTT

HumancDNA ------------------------------------------------------------

ZebrafcDNA ------------------------------------------------------------

FugucDNA ------------------------------------------------------------

Zebrafgene ------------------------------------------------------------

Fugugene GTATAATAGTTTCCAGGATTTTTAAATCAATTGTGCATCATTTCCTGTTGAAAGACGGAT

Smeltgene ------------------------------------------------------------

Herringgene GGTCTCATGCTAAAGTTGCCCCT-------------------------------------

HumancDNA ------------------------------------------------------------

ZebrafcDNA ------------------------------------------------------------

FugucDNA ------------------------------------------------------------

Zebrafgene -----------------------------------------TTTAGGGACTGTTACTTAA

Fugugene TTACGATTAAGTGTGATACATTTTTGCAGTGTGTTTATGTATCTAATCGTATTTAATCAT

Smeltgene ------------------------------------------------------------

Herringgene ---------------------------------------GTTTTATTTATTTATTTATTC

HumancDNA ------------------------------------------------------------

ZebrafcDNA --------GTTCCTGGGCTTCCAACTCCCATCGAGAACATGATTCTTCGTTACGTGAAGG

FugucDNA --------GTGCCAGGCCTTCCCACTCCTATTGAGAACATGATCTTGCGCTACGTCAAGG

Zebrafgene TTTTTCAGGTTCCTGGGCTTCCAACTCCCATCGAGAACATGATTCTTCGTTACGTGAAGG

Fugugene CCATACAGGTGCCAGGCCTTCCCACTCCTATTGAGAACATGATCTTGCGCTACGTCAAGG

Smeltgene ------------------------------------------------------------

Herringgene CTCCCCAGGTCCCAGGCCTTCCCACTCCAATTGAGAATATGATTCTCCGCTACGTGAAAG

HumancDNA --------GTCCCTGGGCTGCCGACGCCCATAGAGAATATGATCCTTCGATACGTGAAGG

ZebrafcDNA CTAAAGCTGACTGGTGGACCAACACGG

FugucDNA CAAAAGCTGACTGGTGGACAAATACCG

Zebrafgene CTAAAGCTGACTGGTGGACCAACACGG

Fugugene CAAAAGCTGACTGGTGGACAAATACCG

Smeltgene ---------------------------

Herringgene CC-------------------------

HumancDNA CCAAGGCTGACTGGTGGACCAACACTG

The Danio Prp8p genomic sequence can be obtained from the Genbank nucleotide database under accession number CR522882.5. The complete coding sequence spans nucleotides 88436-76081.

The Fugu genomic sequence can be found at <http://fugu.hgmp.mrc.ac.uk/fugu-bin/clonesearch> under accession number M000247 (scaffold 247). The complete coding sequence spans bases 218952-231155.

The record for human Prp8p can be retrieved from the gene database at Genbank using accession number 10594. The cDNA sequence has accession number NM_006445.

The herring Prp8p genomic sequence can be obtained from the Genbank nucleotide database under accession number DQ008166.

The smelt Prp8p genomic sequence can be obtained from the Genbank nucleotide database under accession number DQ008165.
