# Supplementary material for: Lateral Transfer of a Lectin-Like Antifreeze Protein Gene in Fishes
Source: PLoS One. 2008 Jul 9;3(7):e2616. doi: 10.1371/journal.pone.0002616 (PMC2440524; doi:10.1371/journal.pone.0002616)
Supplement: Table S1 — Estimated numbers and rates of synonymous and non-synonymous substitutions for the type II AFPs and Prp8p coding sequences of selected fish, including Rainbow smelt (Smelt) and Japanese Smelt (JpSmelt). (0.05 MB DOC) [file pone.0002616.s003.doc]

**Supporting Table 1:** Estimated numbers and rates of synonymous and non-synonymous substitutions for the type II AFPs and Prp8p coding sequences of selected fish, including Rainbow smelt (Smelt) and Japanese Smelt (JpSmelt).

| Sequences Compared | *S_d_^1^* | *S* | *p_S_* | *N_d_* | *N* | *p_N_* | *p_N_/p_S_* | *d_N_/d_S_* |
| --- | --- | --- | --- | --- | --- | --- | --- | --- |
| Type II AFPs*^2^* |  |  |  |  |  |  |  |  |
| Herring vs Smelt | 6.5 | 73.3 | 0.089 | 23.5 | 301.7 | 0.078 | 0.879 | 0.872 |
| Herring vs JpSmelt | 6.5 | 74.5 | 0.087 | 25.5 | 300.5 | 0.085 | 0.973 | 0.971 |
| Smelt vs JpSmelt | 7.0 | 74.8 | 0.094 | 32.0 | 300.2 | 0.107 | 1.140 | 1.151 |
| Sea Raven vs Herring | 30.7 | 71.0 | 0.432 | 101.3 | 283.0 | 0.358 | 0.829 | 0.757 |
| Sea Raven vs Smelt | 31.7 | 70.7 | 0.448 | 96.3 | 283.3 | 0.340 | 0.759 | 0.664 |
| SeaRaven vs JpSmelt | 30.7 | 71.7 | 0.428 | 101.3 | 282.3 | 0.359 | 0.839 | 0.770 |
|  |  |  |  |  |  |  |  |  |
| Prp8p*^3^* |  |  |  |  |  |  |  |  |
| Herring vs Smelt | 75.5 | 157.8 | 0.478 | 6.5 | 559.2 | 0.012 | 0.024 | 0.015 |
| Herring vs Zebrafish | 94.0 | 157.2 | 0.598 | 7.0 | 559.8 | 0.013 | 0.021 | 0.011 |
| Herring vs Takifugu | 96.0 | 157.7 | 0.609 | 4.0 | 559.3 | 0.007 | 0.012 | 0.006 |
| Smelt vs Zebrafish | 103.0 | 156.3 | 0.659 | 10.0 | 560.7 | 0.018 | 0.027 | 0.011 |
| Smelt vs Takifugu | 87.5 | 156.8 | 0.558 | 2.5 | 560.2 | 0.004 | 0.008 | 0.004 |
| Zebrafish vs Takifugu | 101.0 | 156.2 | 0.647 | 8.0 | 560.8 | 0.014 | 0.022 | 0.010 |

*^1^* Values calculated using the SNAP tool (Korber 2000) by the method of Nei and Gojobori (1986) where *S_d_* = number of synonymous differences, *S* = number of synonymous sites *p_S_* = *S_d_*/*S*, *N_d_* = number of nonsynonymous differences, *N* = number of nonsynonymous sites, *p_N_* = *N_d_*/*N*, and *d_N_/d_S_* is an adjusted estimate of *p_N_/p_S_*. *^2^* Accession numbers or citations are given in materials and methods and the legend of figure 1. *^3^* Accession numbers are given in supporting figure 6.
