# Supplementary material for: Lateral Transfer of a Lectin-Like Antifreeze Protein Gene in Fishes
Source: PLoS One. 2008 Jul 9;3(7):e2616. doi: 10.1371/journal.pone.0002616 (PMC2440524; doi:10.1371/journal.pone.0002616)
Supplement: Table S2 — Comparison of the number of times each codon is found in the type II AFP genes and the cloned portion of the Prp8p genes. (0.15 MB DOC) [file pone.0002616.s004.doc]

**Supporting Table 2: Comparison of the number of times each codon is found in the type II AFP genes and the cloned portion of the Prp8p genes.**

|  |  | Type II AFPs | | | Prp8p | |
| --- | --- | --- | --- | --- | --- | --- |
| Codon*^1^* | Amino Acid | Sea Raven | Herring | Smelt | Herring | Smelt |
| TTT | Phe |  | 3 |  | 2 |  |
| TTC |  |  | 2 |  | 11 a |  |
|  |  |  |  |  |  |  |
| TCT | Ser |  | 2 |  | 1 |  |
| TCC |  |  | 1 |  | 5 |  |
| TCA |  |  | 4 |  | 0 |  |
| TCG |  |  | 0 |  | 0 |  |
| AGT |  |  | 1 |  | 0 |  |
| AGC |  |  | 3 |  | 3 |  |
|  |  |  |  |  |  |  |
| TAT | Trp |  | 1 |  | 1 |  |
| TAC |  |  | 0 |  | 10 |  |
|  |  |  |  |  |  |  |
| TGT | Cys |  | 5 |  | 1 |  |
| TGC |  |  | 6 |  | 4 |  |
|  |  |  |  |  |  |  |
| TTA | Leu |  | 3 |  | 0 |  |
| TTG |  |  | 1 |  | 2 |  |
| CTT |  |  | 4 |  | 3 |  |
| CTC |  |  | 1 |  | 8 |  |
| CTA |  |  | 1 |  | 1 |  |
| CTG |  |  | 4 |  | 20 |  |
|  |  |  |  |  |  |  |
| CCT | Pro |  | 2 |  | 2 |  |
| CCC |  |  | 1 |  | 3 |  |
| CCA |  |  | 3 |  | 4 |  |
| CCG |  |  | 0 |  | 1 |  |
|  |  |  |  |  |  |  |
| CAT | His |  | 3 |  | 3 |  |
| CAC |  |  | 1 |  | 7 |  |
|  |  |  |  |  |  |  |
| CGT | Arg |  | 1 |  | 4 |  |
| CGC |  |  | 1 |  | 5 |  |
| CGA |  |  | 0 |  | 1 |  |
| CGG |  |  | 0 |  | 0 |  |
| AGA |  |  | 0 |  | 7 |  |
| AGG |  |  | 0 |  | 4 |  |
|  |  |  |  |  |  |  |
|  |  |  |  |  |  |  |
| CAA | Gln |  | 3 |  | 0 |  |
| CAG |  |  | 2 |  | 12 |  |
|  |  |  |  |  |  |  |
| ATT | Ile |  | 3 |  | 5 |  |
| ATC |  |  | 3 |  | 5 |  |
| ATA |  |  | 2 |  | 1 |  |
|  |  |  |  |  |  |  |
| ACT | Thr |  | 5 |  | 2 |  |
| ACC |  |  | 3 |  | 5 |  |
| ACA |  |  | 4 |  | 2 |  |
| ACG |  |  | 2 |  | 2 |  |
|  |  |  |  |  |  |  |
| AAT | Asn |  | 4 |  | 2 |  |
| AAC |  |  | 1 |  | 11 |  |
|  |  |  |  |  |  |  |
| AAA | Lys |  | 2 |  | 3 |  |
| AAG |  |  | 5 |  | 18 |  |
|  |  |  |  |  |  |  |
| GTT | Val |  | 3 |  | 2 |  |
| GTC |  |  | 0 |  | 5 |  |
| GTA |  |  | 0 |  | 0 |  |
| GTG |  |  | 2 |  | 11 |  |
|  |  |  |  |  |  |  |
| GCT | Ala |  | 7 |  | 3 |  |
| GCC |  |  | 2 |  | 6 |  |
| GCA |  |  | 4 |  | 0 |  |
| GCG |  |  | 0 |  | 2 |  |
|  |  |  |  |  |  |  |
| GAT | Asp |  | 5 |  | 1 |  |
| GAC |  |  | 5 |  | 8 |  |
|  |  |  |  |  |  |  |
| GGT | Gly |  | 2 |  | 1 |  |
| GGC |  |  | 1 |  | 10 |  |
| GGA |  |  | 3 |  | 5 |  |
| GGG |  |  | 1 |  | 3 |  |
|  |  |  |  |  |  |  |
| GAA | Glu |  | 3 |  | 1 |  |
| GAG |  |  | 4 |  | 9 |  |

*^1^* Codon usage is only shown for amino acids that are observed at least 5 or 10 times for 2-fold and 4-fold degenerate sites in the AFP sequences respectively.

Yellow, often mistranslated so appear to be rarer.

Green, the complement of stop codons are rarer in coding sequences.
